# Sequence-encoded hexagonal lattices in multichannel peptide nanofibrils

**DOI:** 10.1038/s41586-026-11016-2

**Published:** 2026-09-23

**Authors:** Jasmina Gačanin, Francesca Mazzotta, Luis Andre Baptista, Nikolay Stoyanov, Matthias Schmidt, Nico Alleva, Thunchanok Thummaraj, Fanny Bonnicel, Cong Zhou, Lei Gao, Jan Münch, Mischa Bonn, Marcus Fändrich, Ingo Lieberwirth, Robinson Cortes-Huerto, Katharina Landfester, Tanja Weil

**Affiliations:** 1 Max Planck Institute for Polymer Research, Mainz, Germany; 2 Institute of Inorganic Chemistry I, Ulm University, Ulm, Germany; 3 Institute of Protein Biochemistry, Ulm University, Ulm, Germany; 4 Institute of Molecular Virology, Ulm University Medical Center, Ulm, Germany

**Keywords:** Supramolecular chemistry, Self-assembly, Molecular self-assembly, Materials science, Structural biology

## Abstract

Structural complexity in biological matter arises from molecular information that encodes supramolecular assembly across length scales^1–3^. Here we show that minimal nine-residue peptides can encode discrete lateral interaction motifs that direct supramolecular organization. These motifs generate hexagonal pores and hierarchically tile into multichannel nanofibrils with defined topology. Sequence-encoded amphiphilicity combines a cross-β-dimer, an inversion point and a trimeric junction to create complementary interfaces that couple lateral growth to axial stacking, yielding honeycomb lattices with continuous approximately 5-nm solvent-accessible nanochannels. Cryo-electron microscopy resolves the supramolecular architecture and shows that lattice symmetry and pore geometry are preserved across variants. Systematic perturbations establish sequence–structure rules linking residue position to supramolecular symmetry, lattice propagation and channel topology. Molecular dynamics simulations and vibrational spectroscopy show that the channels remain water accessible and show sequence-tunable hydration. These findings establish that a minimal, sequence-encoded interaction hierarchy can programme long-range supramolecular order, providing a general framework for how short peptides can encode complex, symmetry-defined architectures^4–12^.

## Abstract (web summary)

Nine-residue peptides can encode discrete interaction motifs that direct the formation of hexagonal pores, which hierarchically tile into laterally expandable multichannel nanofibrils with defined topology.

## Main

Biological materials derive functional complexity from molecular information that encodes their three-dimensional structure and hierarchical organization across length scales. Polypeptides offer a rich chemical design space^13–15^, and β-sheet-rich amyloid fibrils represent one of their most prevalent supramolecular states, with roles ranging from hormone storage to pathological aggregation^16–18^. Recent advances in cryo-electron microscopy (cryo-EM) have revealed these assemblies at high resolution, resolving their atomic structures in the hydrated state^17,18^, and disclosing the packing and polymorphism of cross-β-motifs.

**Extended Data Fig. 1:**
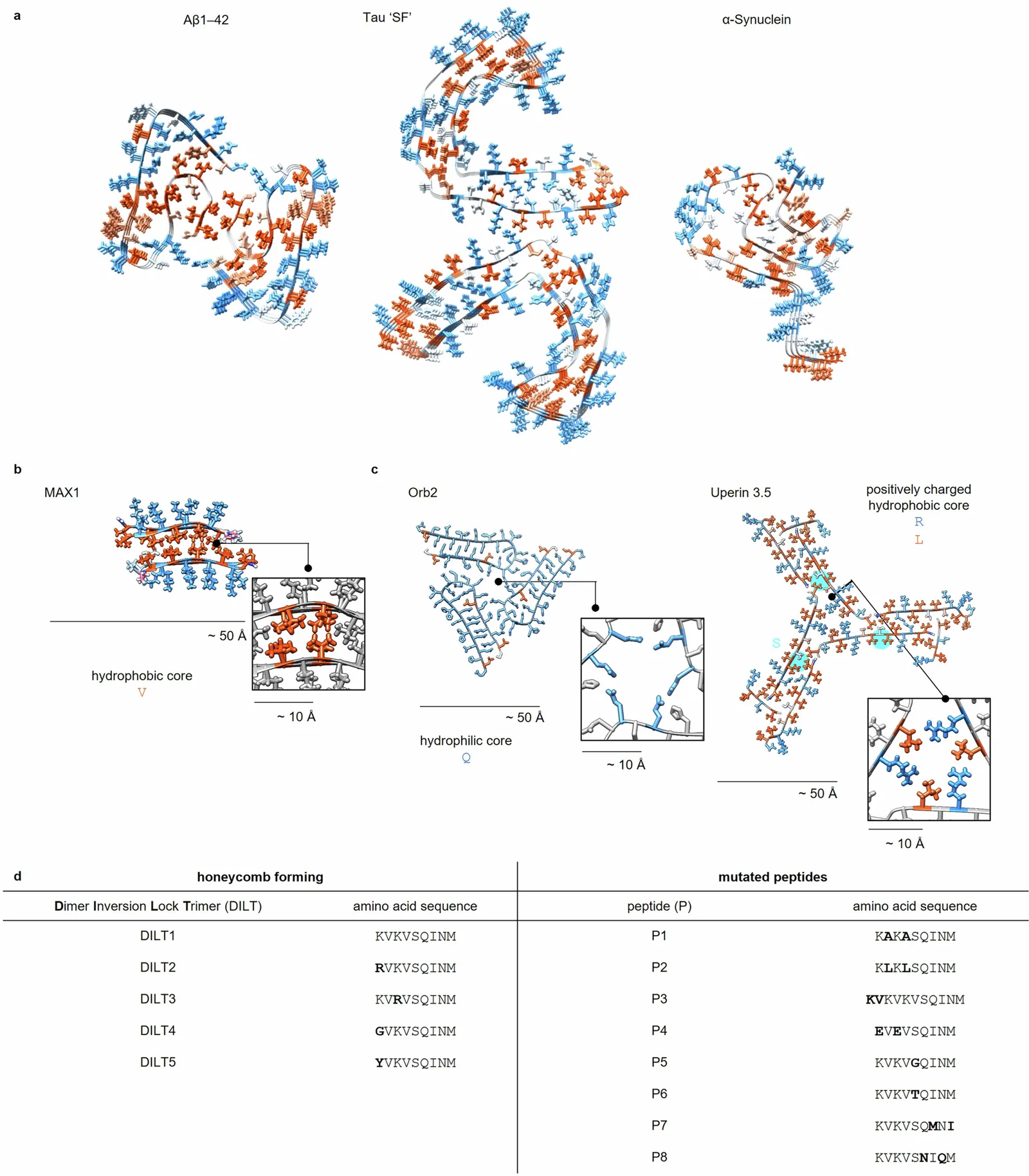
Representative amyloid fibrils, Dimer Inversion Lock Trimer (DILT)-related cross-β dimer and trimer motifs, and summary of the DILT and control peptide libraries. **a,** Atomic structures of classical amyloids Aβ1–42 (PDB: 5OQV)^18^, tau ‘SF’ (PDB: 5O3T)^17^ and α-synuclein (PDB: 2N0A)^73^. **b**, Atomic model of the cross-β-sheet forming peptide MAX1 (PDB: 2N1E)^35^. **c,** Atomic models of the trimeric junction-forming functional amyloids Orb2 (PDB: 6VPS)^37^ and Uperin 3.5 (PDB: 7QV5)^38^. Structures in a–c are colour-coded by amino-acid hydrophilicity (blue, hydrophilic; orange, hydrophobic) according to the Kyte–Doolittle hydrophobicity scale^74^. Insets in b and c highlight the respective cores. Scale bars were generated in Chimera and should be regarded as approximate owing to the three-dimensional rendering of the structures. **d**, Dimer - Inversion - Lock - Trimer peptide library and systematic screening matrix of honeycomb-forming (DILT1 – DILT5) and mutated peptide (P1 – P8) amino acid sequences.

Most peptide assemblies elongate as one-dimensional fibrils whose lateral interfaces are polymorphic and therefore difficult to design, preventing controlled tiling into higher-dimensional lattices (Extended Data Fig. 1a). Programmable hierarchical growth has been achieved in coiled-coil proteins^19–26^ and collagen-mimetic systems through large pre-organized interfaces^27,28^, whereas peptide nanochannels formed from cyclic scaffolds rely on covalent backbone pre-organization^29–34^. By contrast, controlled two- or three-dimensional lattice formation from minimal linear β-sheet peptides is rare^5–12^. Consequently, a geometry-guided framework for programmable multichannel architectures from short linear β-sheet sequences has remained elusive.

**Fig. 1:**
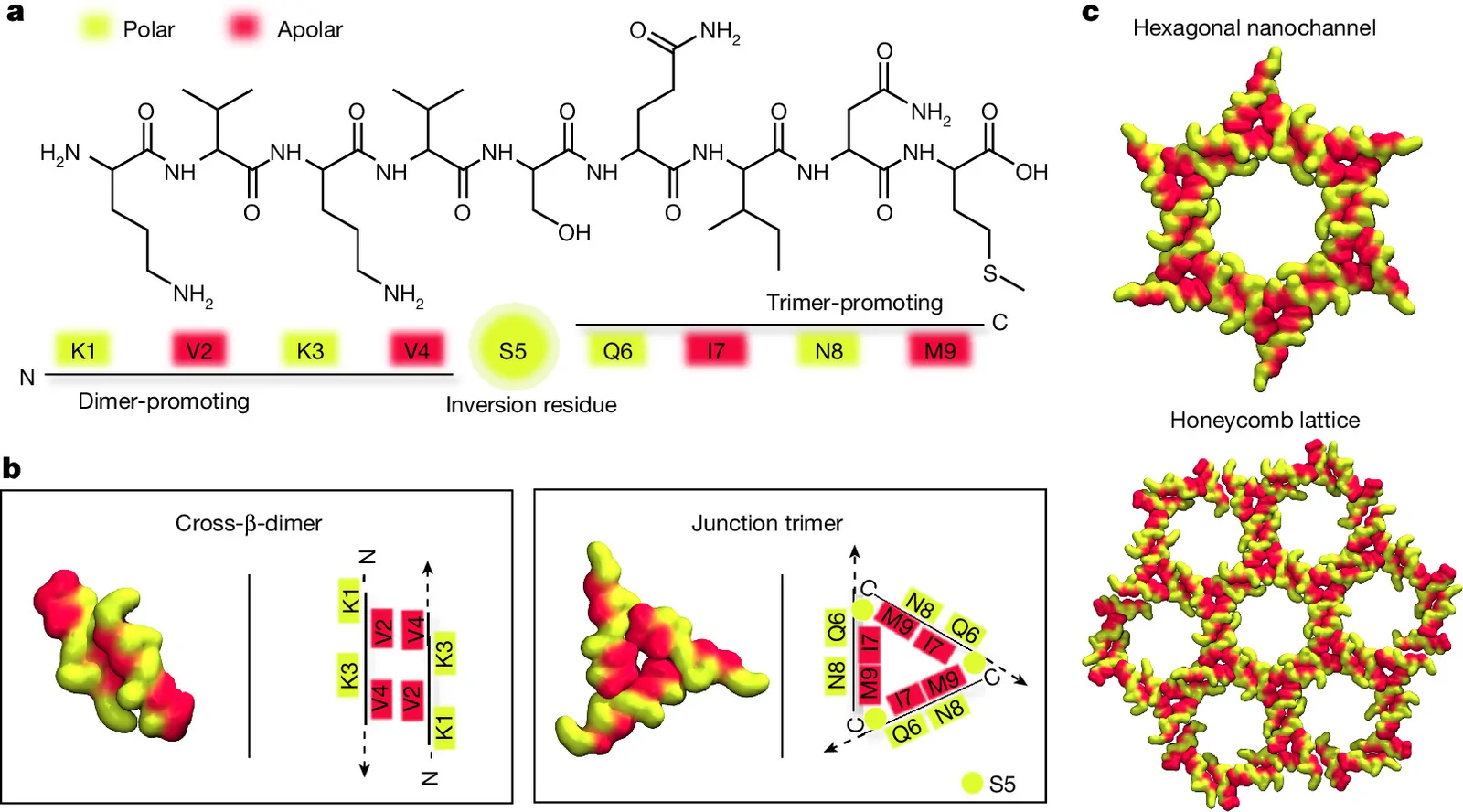
Sequence-encoded geometry of a hexagonal peptide lattice. **a**, Modular design of the DILT peptide family showing the cross-β-dimer-forming interface, the inversion residue and the trimeric-junction-forming interface that together encode complementary axial and lateral interfaces. Polar and apolar surface potentials are shown in yellow and red, respectively. The yellow sphere denotes the inversion residue. **b**, Cross-β-dimers define the principal β-sheet stacking and association interface, whereas trimer junctions provide lateral connectivity through a shared hydrophobic core and polar stabilizing interactions. **c**, Coupling of dimer stacking and junction formation generates a hexagonal pore that propagates along the fibril axis to form a hexagonal nanochannel and supports lateral growth into continuous multichannel honeycomb lattices that preserve pore geometry.

Here we show that amphiphilic nonapeptides encode discrete interaction motifs that direct the formation of hexagonal pores, which hierarchically tile into laterally expandable multichannel nanofibrils: a cross-β-dimer that mediates lateral and axial growth, a trimeric junction that encodes lateral connectivity and symmetry, and a central inversion residue that orients the two motifs (Fig. 1a) to create complementary interfaces that couple lateral organization to axial stacking. Cryo-EM, sequence perturbation, molecular dynamics simulations and vibrational spectroscopy reveal how these motifs couple axial stacking, lateral tiling and sequence-dependent channel hydration. Together, these results show that minimal sequence-encoded interaction motifs can programme supramolecular symmetry and hierarchical lattice growth, enabling multichannel peptide nanofibrils from short linear sequences.

## Sequence-encoded hexagonal lattice

The dimer–inversion–lock trimer (DILT) sequence family encodes an interaction geometry enabling hexagonal nanochannel symmetry and honeycomb lattice formation upon assembly. This nine-residue peptide design combines a cross-β-dimer-forming segment, a central inversion residue and a trimeric junction (Fig. 1a and Supplementary Fig. 1). In the prototype DILT1 (K1–V2–K3–V4–S5–Q6–I7–N8–M9), the N-terminal amphiphilic K1–V2–K3–V4 motif promotes face-to-face cross-β-pairing with hydrophobic packing, thereby defining hydrophobic contact surfaces for lateral organization and an axial stacking interface through backbone hydrogen bonding. This segment was inspired by the cross-β-forming oligopeptide MAX1 family^35^ (Extended Data Fig. 1b). The C-terminal Q6–I7–N8–M9 segment encodes a trimeric interaction, in which polar side chains provide directional hydrogen bonds and hydrophobic residues form a shared core. This junction-forming segment was based on the native HIV glycoprotein (gp120)-derived amyloidogenic sequence and related synthetically optimized, functional cross-β-forming oligopeptides^36^. The design of the trimeric junction in the DILT family adapts the physicochemical logic observed in naturally occurring trimeric amyloids. Tightly interdigitated residues generate a flat, propeller-shaped geometry, amide-based residues mediate hydrophilic interfaces and directional hydrogen bonding in solvated polar topologies, as in Orb2^37^, and serine terminates the trimeric core by interrupting the hydrophobic interface between tightly arranged β-sheets (as in uperin3.5)^38^ (Extended Data Fig. 1c). In DILT, the re-orientation of these two interaction motifs, the KVKV β-sheet and the QINM junction, via a central serine acting as an inversion element, achieves the necessary spatial arrangement of complementary axial and lateral interfaces to yield hierarchical tiling with hexagonal fibril symmetry without requiring a cyclic backbone or larger pre-organized scaffold. This modular arrangement transforms the intrinsically one-dimensional growth of β-sheets into a symmetry-defined three-dimensional (3D) architecture: cross-β-pairing drives fibril growth, whereas the trimeric junction positions neighbouring strands at defined angles, yielding a hexagonal pore that propagates symmetry along the fibril axis (Fig. 1b,c). Together, the cross-β-dimer and trimeric junction define the hexagonal geometry of a continuous fibrillar nanochannel. Conservative and non-conservative mutations were then used to perturb charge distribution, hydrophobic packing and junction geometry while retaining the overall modular framework (Fig. 1a and Extended Data Fig. 1d), highlighting elements of the encoded geometry that are required for hexagonal lattice formation.

## Cryo-EM resolves the hexagonal lattice

**Fig. 2:**
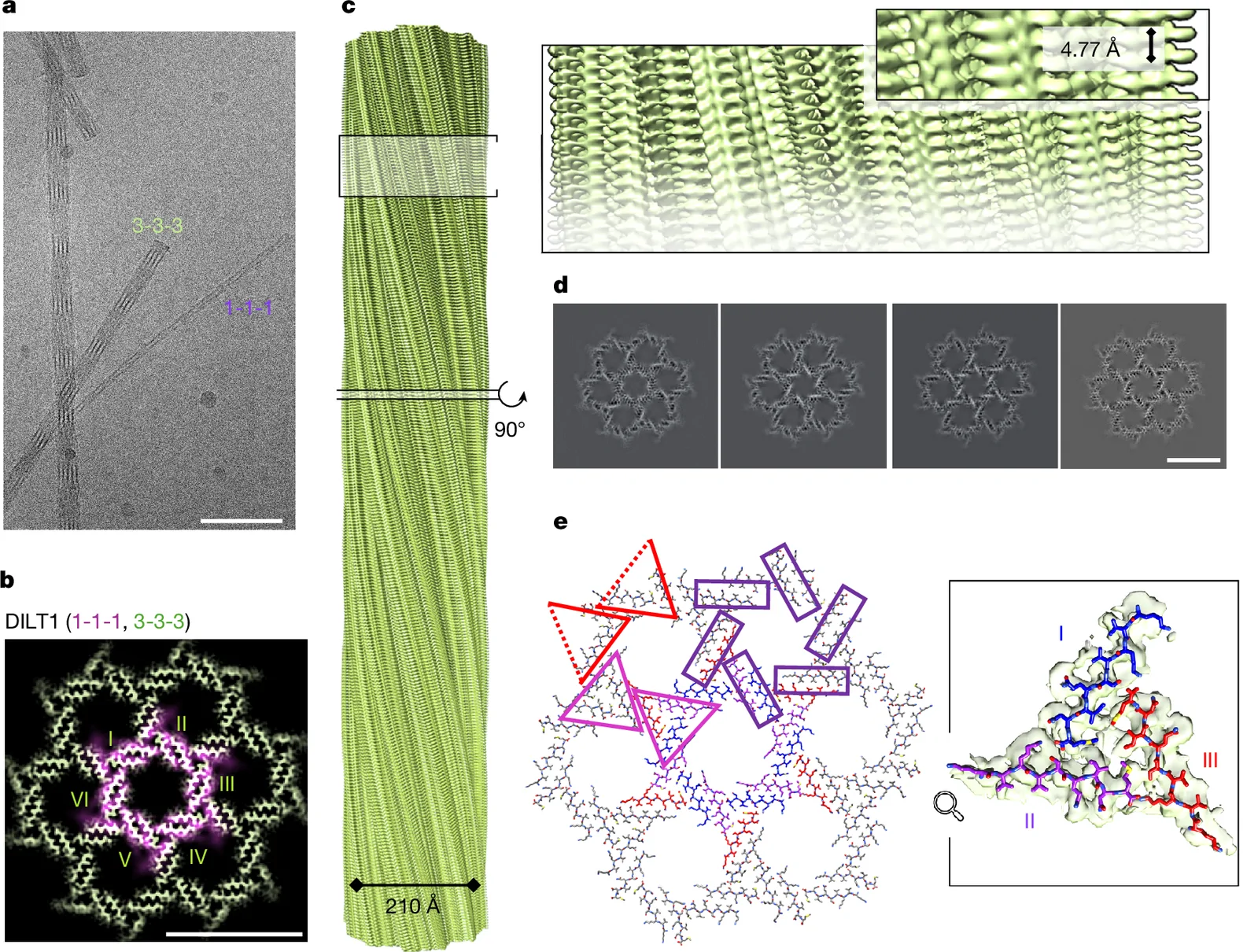
Cryo-EM structure of a hexagonal nanochannel fibril. **a**, Cryo-EM micrograph of DILT1 showing single-channel (1-1-1) and predominant honeycomb (3-3-3) fibril morphologies with a characteristic, intermittent striped pattern. **b**, Reconstructed cross-sections of DILT1 revealing six face-to-face packed cross-β-dimers arranged around a central cavity with *C*_6_ symmetry and six (I–VI) junction-forming chains. **c**, Density map of the DILT1 3-3-3 honeycomb fibril highlighting the 4.77 Å β-strand stacking along the fibril axis. **d**, Consecutive two-dimensional slices through the electron density of the DILT1 3-3-3 reconstruction showing chain directionality and lattice symmetry. **e**, Atomic model of the DILT1 3-3-3 architecture with 60 peptide chains per cross-section. Insets: the cross-β-dimer (rectangles) and trimeric-junction interfaces (triangles) that mediate axial propagation and lateral growth, including a close-up of the trimeric junction formed by three DILT1 peptide chains. Scale bars, 100 nm (**a**), 100 Å (**b**,**d**).

**Extended Data Fig. 2:**
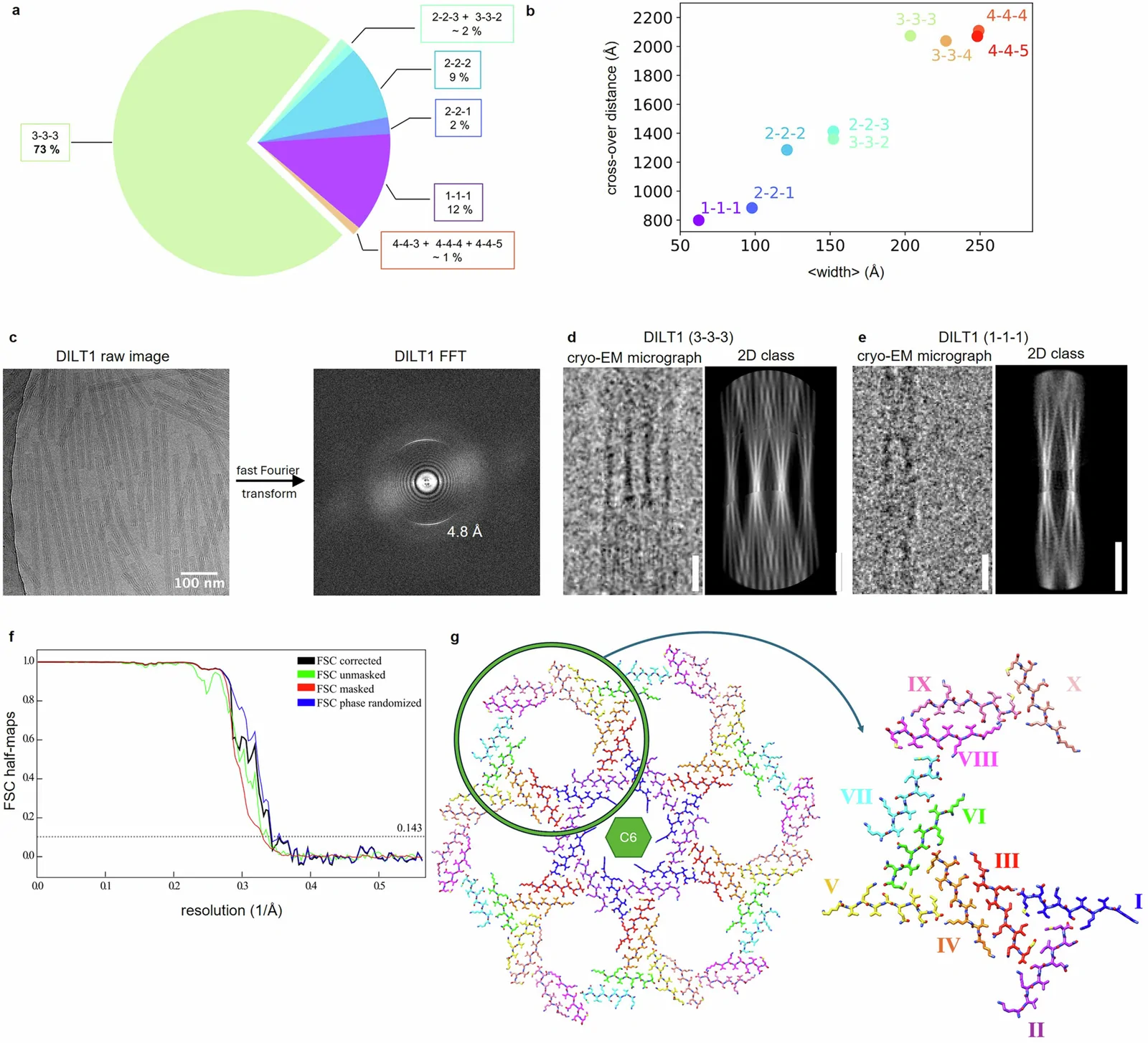
Isomorphs, cryo-EM analysis and atomic model of DILT1 (3-3-3). **a**, Relative abundance of the nine detected DILT1 isomorphologies quantified in 100 cryo-EM micrographs after 53 days of incubation. **b**, Average width and fibril crossover distance of detected DILT1 isomorphologies. **c**, Single-particle cryo-EM micrograph with corresponding fast Fourier transformation (FFT) of DILT1 showing the diffraction pattern of the image. In the diffraction pattern, the 4.8 Å feature characteristic of amyloid structure is highlighted by the bright signal^75^. **d**,**e**, Cryo-EM micrographs enlarged to highlight the fibril pattern, shown alongside combined 2D classes of the 3-3-3 and 1-1-1 morphologies. Combining multiple 2D classes enables visualization of the full fibril. The figure highlights the unresolved and striped patterns and shows how the single-particle approach improves signal-to-noise and facilitates interpretation of the cryo-EM micrographs. **f**, Fourier shell correlation (FSC) curves of the two half-maps of the reconstruction calculated for the DILT1 3-3-3 honeycomb fibrils obtained from the RELION postprocess job. Black, FSC corrected; green, FSC unmasked maps; red, corrected FSC phase-randomized masked maps; blue, FSC masked maps. **g**, Atomic model of the DILT1 3-3-3 honeycomb fibril colour-coded according to the fundamental chains. The fibril has C6 symmetry, which enables the description and nomenclature of the chain IDs. Chains I (blue), II (purple) and III (red) form the inner hexagon, are atomically modelled, and were used for the MD simulations. The additional chains (IV to X) were generated by propagating the three fundamental chains into the outer-hexagon density. In Fig. 2 of the main text, these chains are shown in grey. Chain IV (orange) forms the cross-β interaction with chain III (red) and bridges to the external hexagon by forming the junction with chains V (green) and VI (yellow). To close the external hexagon, chain VII (cyan) forms a cross-β interaction with chain V (green) and a semi-junction with chain VIII (magenta). Chain VIII then forms a cross-β interaction with chain IX (pink), which in turn forms a semi-junction with chain X (salmon pink). Chain X can then connect with a symmetry-related copy of chain VI (yellow).

Cryo-EM resolves the encoded interaction geometry as a hexagonal β-sheet lattice containing a continuous central nanochannel. DILT1 forms highly ordered fibrils that adopt two principal structural isomorphs: a single-channel hexagonal architecture (1-1-1; 12% relative abundance) and a laterally expanded honeycomb lattice (3-3-3; 73% relative abundance) (Fig. 2a and Extended Data Fig. 2a,b). The three-digit notation denotes the number of hexagonal pores in each of the three lattice sectors visible in the cross-section. Helical reconstruction resolved the 1-1-1 and 3-3-3 structures to 4.0 Å and 2.9 Å, respectively, revealing a repeating unit composed of 12 peptide chains arranged as 6 face-to-face packed cross-β-dimers around a central cavity (Fig. 2b–e and Extended Data Fig. 2c–g). In the plane perpendicular to the fibril axis, this unit exhibits *C*_6_ symmetry and encloses an approximately 5-nm central cavity that propagates continuously along the fibril. Successive layers therefore preserve pore geometry along the fibril axis, defining a continuous hexagonal channel. In the single-channel morphology (1-1-1), the peripheral density connects adjacent dimers through partially resolved triangular features consistent with predicted trimeric junctions. In the predominant honeycomb architecture, these junctions are fully resolved and mediate symmetry and lateral growth at the lattice perimeter (chains I–VI, Fig. 2b). This lateral tiling yields 7 hexagons per layer and a total of 60 peptide chains in a single cross-section.

The clear axial peptide stacking at 4.7–4.8 Å and well-resolved electron density in the higher-resolution reconstruction enable atomic modelling of the complete lattice and assignment of the principal dimer and junction interfaces. The atomic model of the 3-3-3 fibril yields a diameter of 210 Å, a helical rise of 4.77 Å and a twist of −0.64°. Application of *C*_6_ symmetry reduces the asymmetric unit to ten unique chains per layer, three of which were built directly into the density and propagated by symmetry to generate the full architecture (Extended Data Fig. 2g). The peripheral regions show weaker electron density, indicating local dynamics or incompletely formed junctions, which account for the semi-triangular features observed in the 1-1-1 morphology. The absence of the third junction-forming chain reflects insufficiently defined cryo-EM density to model its sequence, although the residual density is consistent with its presence. Thus, the 1-1-1 and 3-3-3 morphologies represent isomorphs of the same underlying lattice geometry that differ in the extent of lateral expansion, rather than distinct assembly modes. Helical torsion decreases with fibril diameter: fibrils containing more hexagons show larger crossover distances and smaller twist angles (Extended Data Fig. 2b).

## Interaction hierarchy of the hexagonal lattice

**Fig. 3:**
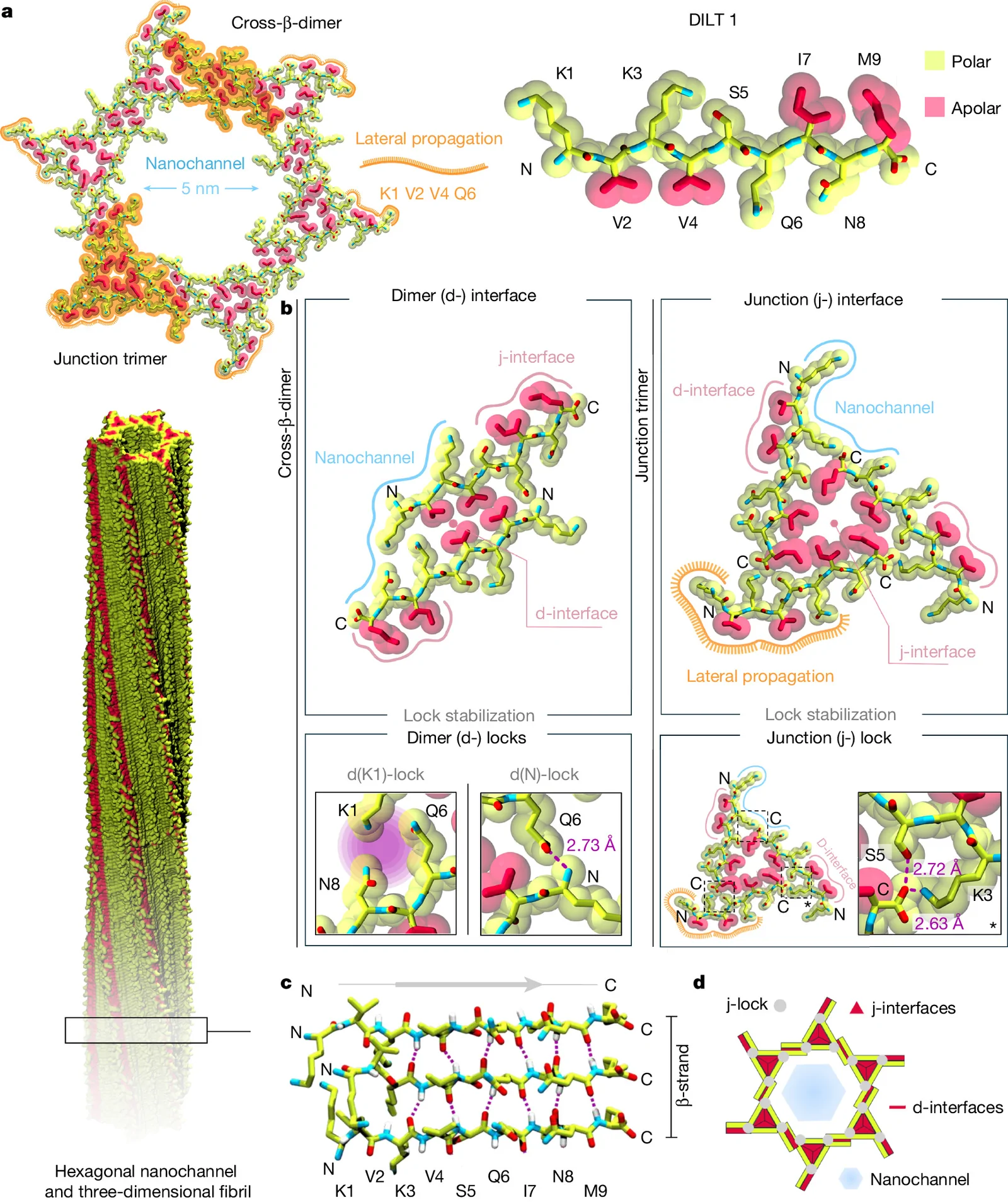
Interaction hierarchy linking axial propagation and symmetry-defined lateral growth. **a**, Nanochannel with hexagonal lattice formed by face-to-face packed cross-β-dimers connected through trimeric junctions via lateral propagation sites. Amphiphilic residues of the linear DILT1 sequence map onto polar, pore-lining and hydrophobic interfaces, generating an approximately 5-nm nanochannel within the fibril lattice. **b**, Complementary interaction motifs and representative locking interactions. The d-interface between cross-β-dimers mediates hydrophobic packing for lateral fibril propagation. The j-interface, a trimeric junction, links neighbouring dimers encoding the lateral connectivity for hexagonal lattice formation. The d-locks stabilize lateral registry through polar inter-strand interactions, including hydrogen bonding between Q6 and the N-terminus (d(N)-lock) and interactions involving K1, N8 and Q6 (d(K1)-lock). The j-lock interactions stabilize the junction interface at its tips by directional hydrogen bonds and electrostatic interactions involving S5, K3 and the C-terminus. **c**, Axial stacking of the DILT peptides propagates the lattice along the fibril axis via peptide backbone hydrogen bonds. **d**, Summary of the interaction hierarchy. The j-interfaces (red triangles) provide lateral connectivity and six-fold symmetry, the d-interfaces (red rectangles) mediate β-sheet stacking and lateral association, and the j-locks (grey spheres) reinforce junction cohesion, together supporting a hexagonal nanochannel architecture.

**Extended Data Fig. 3:**
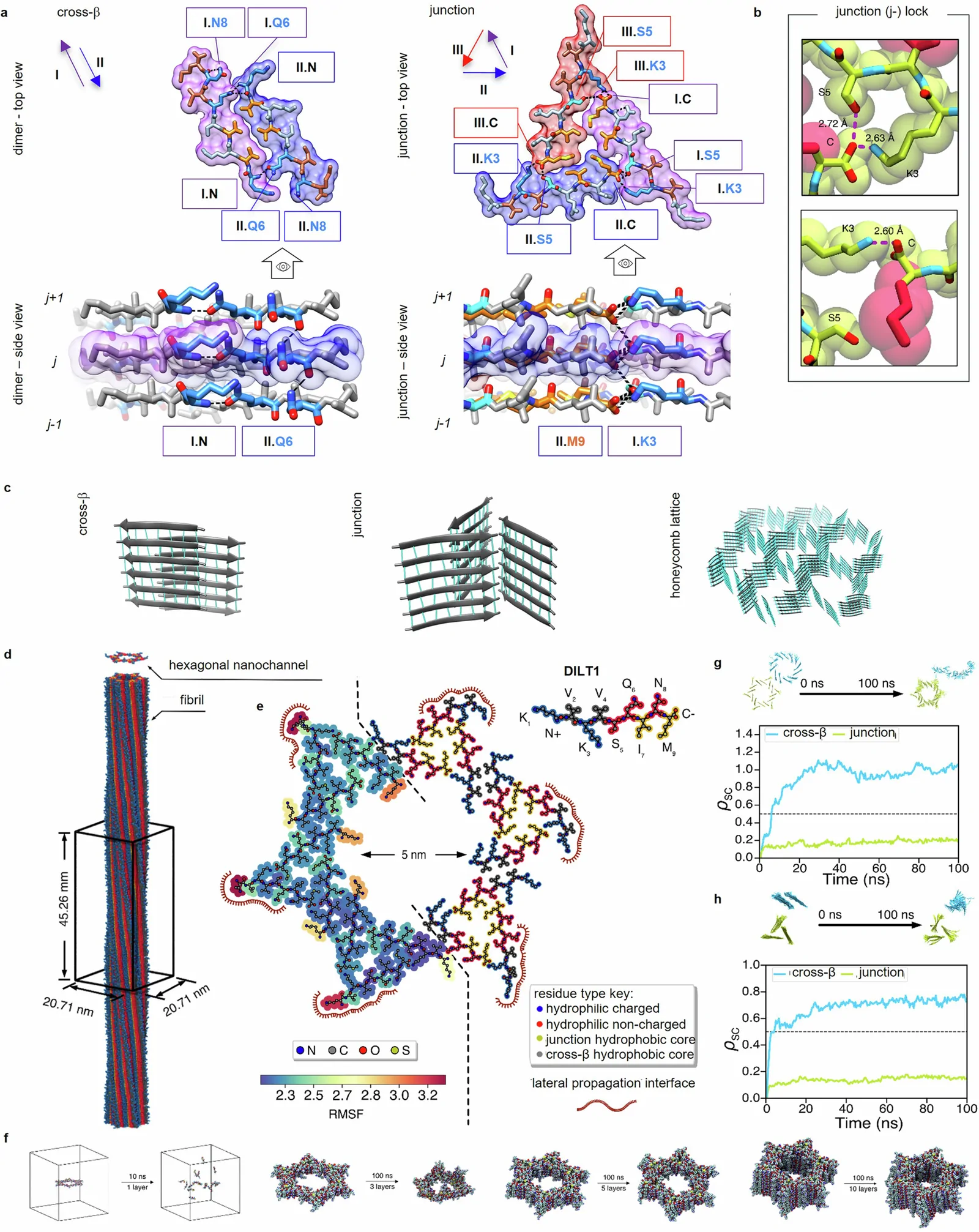
Cryo-EM atomic model of DILT1 (3-3-3) interactions and molecular dynamics integrity analysis of DILT1 fibril constituents. **a**, Cryo-EM atomic model visualising the main interaction motifs. **b**, Molecular dynamics simulation of the junction (J-) lock. Top, lock arrangement between K3 and the C-terminus and the S5-to-C-terminus hydrogen bond. Bottom, K3-to-C-terminus hydrogen bond. **c**, Cryo-EM-derived schematic highlighting axial stacking of DILT1 chains in the dimer and junction motifs, as well as in the honeycomb lattice. **d**, Hexagonal nanochannel and explicit periodic representation of the fibril after 100 ns of molecular dynamics simulation. The orthorhombic simulation box is shown for reference. **e**, Left, average root mean square fluctuation (RMSF) per residue averaged along the fibril axis. Right, conformation of the junction (hydrophobic core residues shown in yellow) and cross-β-sheet (hydrophobic core residues shown in grey) motifs composing the hexagon. The layer also shows the relative position of the lateral propagation site formed by residues K1, V2, V4 and Q6. **f**, Molecular dynamics simulations show that the minimum size of the persistent hexagonal nanochannel consists of five layers. **g**, Integrity of the hexagonal nanochannel. Top, top-view snapshots of hexagon structures with five layers at t = 0 ns and t = 100 ns. Hexagons with (green) and without (blue) a junction are shown. Bottom, scaled dissimilarity index (ρ_SC) for these simulations. **h**, Integrity of the dimeric cross-β-sheet and trimeric junction motifs. Top, top-view snapshots at t = 0 ns and t = 100 ns of the cross-β-sheet (blue) and junction (green) simulations, each with 15 layers. Bottom, scaled dissimilarity index (ρ_SC) of the backbone-atom positions obtained from the molecular dynamics trajectory with five layers.

To define the interaction network associated with the hexagonal lattice, we performed molecular dynamics simulations based on the cryo-EM structure of the DILT1 3-3-3 architecture. The trajectories reveal a cooperative and spatially constrained set of hydrophobic and hydrogen-bonding interactions that couple axial propagation to lateral connectivity and thereby maintain the lattice geometry over the simulated timescale in aqueous buffer solution (Fig. 3a). Within the cross-β-dimer, face-to-face packed chains are stabilized by hydrophobic packing of the V2 and V4 side chains (d-interface; Fig. 3b and Extended Data Fig. 3a), which defines the lateral growth interface. This packing is reinforced by recurrent inter-chain hydrogen bonds. In the fibril plane, hydrogen bonds between Q6 and the N-terminal amine (d(N)-lock) and polar interactions between N8 and K1 (d(K1) lock) provide additional directional stabilization of the lateral registry. At the junction, three peptide chains form a compact triangular hub in which interdigitated I7 and M9 residues create a densely packed hydrophobic core (j-interface; Fig. 3b and Extended Data Fig. 3a), topologically equivalent to a threefold steric-zipper interaction. Persistent attractive interactions between the M9 C-terminus and the side chains of K3 and S5 of a neighbouring chain (j-lock; Fig. 3b and Extended Data Fig. 3b), including hydrogen bonding and electrostatic contacts, further stabilize this connectivity. Along the fibril axis, backbone hydrogen bonding within the cross-β-architecture drives periodic stacking of the hexagonal layers into an extended nanochannel fibril (Fig. 3c and Extended Data Fig. 3c).

Simulations of a 94-layer fibril (44.8 nm in length; total twist 60.16°) retained the experimental lattice geometry over 100 ns (Extended Data Fig. 3d). Root-mean-square fluctuation (RMSF) analysis showed mean backbone fluctuations below 2.5 Å, supporting structural integrity of the model over this timescale (Extended Data Fig. 3e,f). By contrast, isolated cross-β-dimers and their hexagonal assemblies lacking junctions rapidly destabilize, whereas isolated junction motifs and the hexagonal nanochannels remain intact over the simulated timescale (Extended Data Fig. 3g,h), supporting a model in which the junction provides the principal lateral cohesion required for hexagonal channel formation. The enhanced persistence of the junction is consistent with contributions from both the compact hydrophobic j-interface and the three j-locking interactions, which together reinforce lateral cohesion through combined packing and hydrogen-bonding contacts. Mapping these interactions onto the fibril cross-section reveals pronounced amphiphilic partitioning, with buried hydrophobic cores and hydrophilic surfaces lining the pore. This organization is consistent with a solvent-accessible nanochannel in which solvent-exposed polar residues define the channel environment, whereas hydrophobic interfaces stabilize the lattice. Thus, axial propagation, lateral junction formation, locking interactions and backbone hydrogen bonding form a coupled interaction hierarchy that supports an axially and laterally expandable six-fold-symmetric architecture in aqueous solution.

## Hexagonal topology across variants

**Fig. 4:**
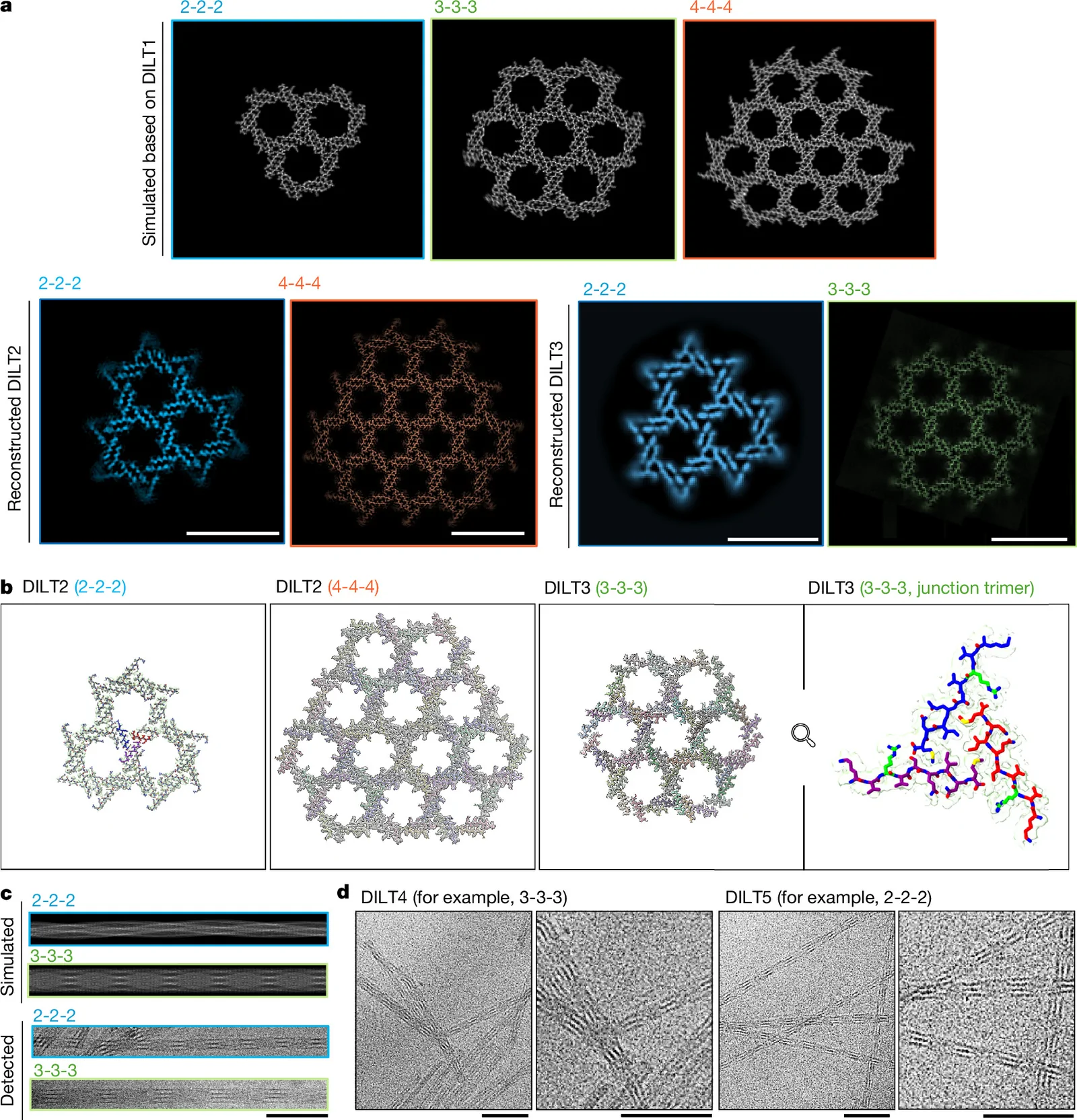
Hexagonal topology across sequence variants. **a**, DILT cross-section matrix. Simulated and reconstructed fibril cross-sections illustrating lateral expansion by integer addition of hexagonal units. **b**, Cryo-EM density maps and atomic models of DILT2 (RVKVSQINM) and DILT3 (KVRVSQINM) showing conserved trimeric junction connectivity and a preserved approximately 5-nm pore diameter. Insets: the three-chain junction motif. **c**, Simulated projections derived from the atomic model reproduce the characteristic intermittent stripe pattern observed in cryo-EM DILT1 micrographs, supporting a common lattice geometry. **d**, Cryo-EM micrographs of DILT4 (GVKVSQINM) and DILT5 (YVKVSQINM) as well as corresponding magnified views show the same stripe patterns as DILT1 (KVKVSQINM), indicating preservation of the hexagonal nanochannel architecture despite sequence variation. The number of stripes correlates with the number of laterally connected hexagons (for example, 3-3-3 or 2-2-2 isomorphs). Scale bars, 100 Å (**a**), 100 nm (**c**,**d**).

**Extended Data Fig. 4:**
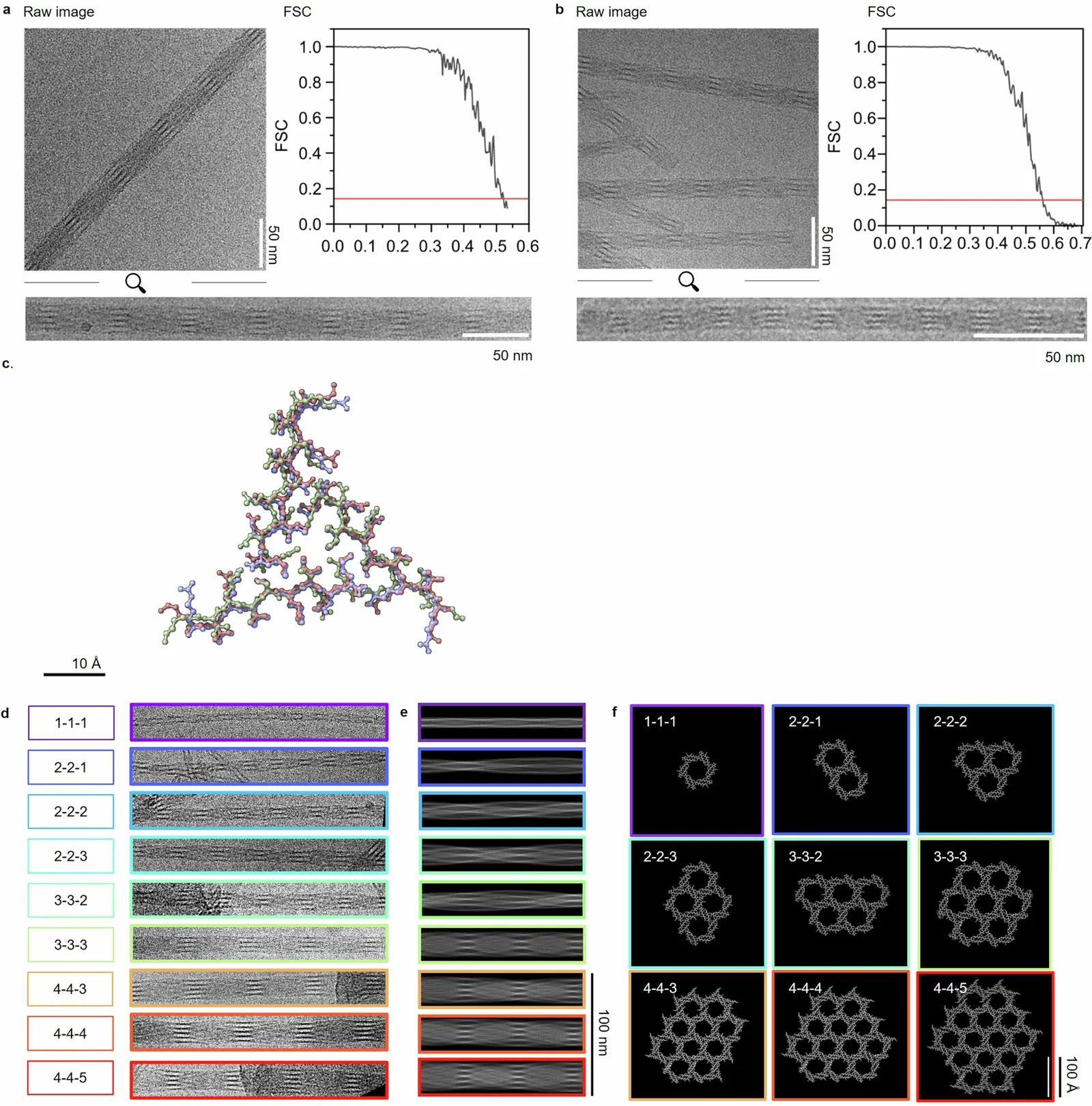
Cryo-EM images and Fourier shell correlation curves of DILT2 and DILT3, and isomorphs of DILT1 fibrils. **a**, Cryo-EM micrograph of DILT2 (4-4-4), together with a close-up view of the fibril and the gold-standard Fourier shell correlation (FSC) curve of the reconstructed DILT2 density. **b**, Cryo-EM micrograph of DILT3 (3-3-3), together with a close-up view of the fibril and the gold-standard Fourier shell correlation (FSC) curve of the reconstructed DILT3 density. **c**, Overlay of the DILT1–3 junction models. DILT1 is shown in green, DILT2 in purple and DILT3 in red. **d**, Detected cryo-EM micrographs of the nine detected DILT1 isomorphs with their respective nomenclature. For example, the 2–2–1 isomorph denotes two double-stripe regions (“2–2”) and a third region with a single stripe (“1”). **e**, Simulated projections and **f**, expected cross-sections of all fibril isomorphs, colour-coded as in d.

To test whether lattice topology is encoded in the peptide sequence, we determined the structures of the arginine variants DILT2 (RVKVSQINM) and DILT3 (KVRVSQINM) using cryo-EM. Both assemble into hexagonal nanochannel architectures with conserved pore geometry and junction connectivity, showing that the lattice organization is preserved across these sequence variants (Fig. 4a). DILT2 predominantly forms a 4-4-4 isomorphology (approximately 80%), with minor 2-2-2 and 1-1-1 populations (approximately 10% each). The first two isomorphologies (4-4-4 and 2-2-2) were reconstructed at 1.9 Å and 3.4 Å resolution, respectively (Fig. 4b and Extended Data Fig. 4a,b). The helical rise and twist were determined to be 4.781 Å and −0.409°, and 4.812 Å and −8.2°, respectively. The symmetry centre depends on the parity of the hexagon arrangement. For odd-numbered hexagon arrangements (for example, 3-3-3), the symmetry axis is located in the centre of the central pore, yielding *C*_6_ symmetry, whereas for even-numbered morphologies (for example, 4-4-4), the symmetry centre is located in the central junction, yielding *C*_3_ symmetry. DILT3 adopts mainly the 3-3-3 morphology (approximately 70%) and was resolved at 1.78 Å resolution, with a helical rise of 4.791 Å and a twist of −0.6115°. At this resolution, side-chain conformations within both the cross-β-dimers and the junction interfaces are directly visualized.

Across all variants, the hexagonal lattice, the trimeric junction and the approximately 5-nm nanochannel remain invariant (Fig. 4b and Extended Data Fig. 4c), showing that global topology emerges from the encoded interaction geometry. Thus, sequence perturbations in these cases modulate local packing without altering the underlying symmetry or pore architecture.

The cryo-EM structures and the molecular-dynamics-derived interaction hierarchy are consistent with lateral growth into additional hexagonal units while maintaining the central pore. Cryo-EM reconstructions support this model by visualizing a family of geometrically related isomorphs that differ in the number of laterally connected hexagons (Fig. 4a,b and Extended Data Fig. 4d–f). These range from single-channel fibrils (1-1-1) to multichannel architectures containing up to 14 pores (4-4-5), comprising several hundred peptide chains in a single cross-section. In all reconstructed cases, the cross-β-registry and pore diameter remain constant, supporting lateral growth by integer tiling of a conserved hexagonal unit rather than by extensive polymorphic reorganization. Simulated projections derived from the atomic model reproduce the intermittent stripe pattern observed in the cryo-EM micrographs. This supports assignment of the fibril pattern to the hexagonal nanochannel architecture of the DILT family. The number of connected hexagons correlates with the characteristic intermittent striped pattern in the cryo-EM images (Fig. 4a,c and Extended Data Fig. 4d–f), providing a morphological fingerprint of the lattice organization. Because this relationship is conserved across DILT1–3, the stripe pattern provides a diagnostic morphological signature that can be used to assess whether mutations preserve hexagonal symmetry.

The related peptides DILT4 (GVKVSQINM) and DILT5 (YVKVSQINM) show the same intermittent stripe pattern, indicating conservation of the hexagonal lattice despite non-conservative substitution at the N-terminal position (Fig. 4d). The sequences of DILT2, DILT4 and DILT5 differ from DILT1 and DILT3 only at residue X1, where K1 is replaced by R, G or Y, suggesting this site as symmetry tolerant and involved in modulating surface chemistry rather than lattice formation. Despite the large variation in fibril width among the detected isomorphs of DILT1–DILT3 (approximately 60–250 Å; Extended Data Fig. 2b), β-strand spacing, pore diameter and junction geometry remain invariant, showing that the encoded interaction geometry constrains the supramolecular topology. The hexagonal nanochannel, therefore, acts as a laterally expandable structural module that supports symmetry-defined multichannel peptide fibrils.

## Sequence constraints of the lattice

**Fig. 5:**
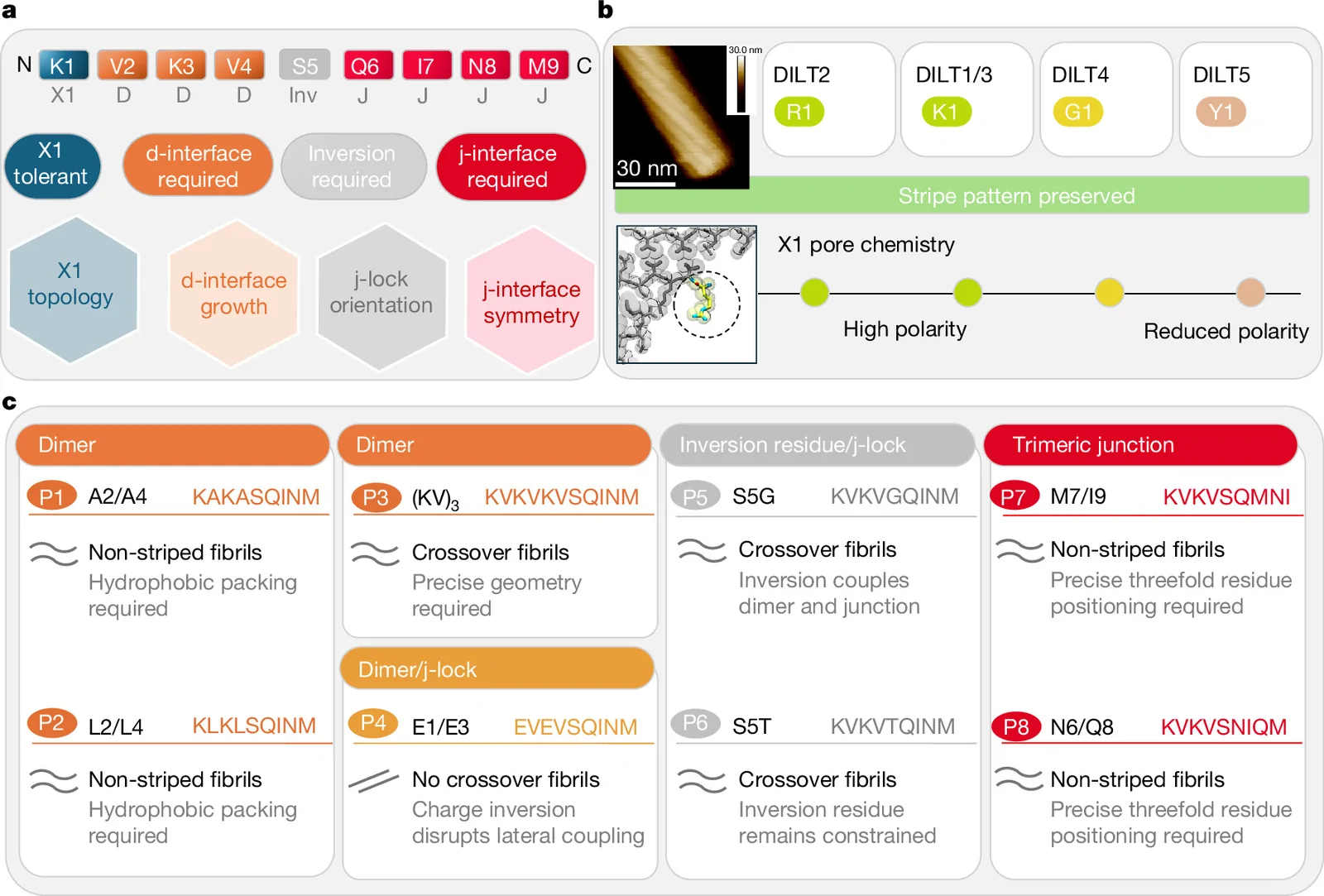
Hierarchical sequence rules define the symmetry-encoded peptide lattice. **a**, Rule map of the DILT1 sequence with symmetry-tolerant site and structurally constrained motifs. Symmetry-tolerant substitutions at the X_1_ position at the N-terminus preserve stripe topology and hexagonal lattice formation. **b**, Tolerated substitutions in DILT1–DILT5 at the X_1_ position preserve the honeycomb lattice and tune fibril topology and pore chemistry. **c**, Peptide mutations with perturbations of required motifs do not produce detectable honeycomb lattices under the matched screening conditions used here. Grouped by targeted motif: disruptions produce crossover and no crossover fibrils without a stripe pattern. Alteration of the hydrophobic dimer interface leads to loss of detectable honeycomb hexagonal lattice formation (P1 and P2). Extension of the dimer motif, or charge substitution within the dimer motif and j-lock, yields non-honeycomb morphologies in the matched screening (P3 and P4).Mutation of the inversion residue and j-lock yields fibrils with crossover lacking lateral organization into the honeycomb hexagonal lattice (P5 and P6). Perturbation of the trimeric junction does not yield detectable hexagonal lattice formation under the tested conditions and produces non-striped fibrils (P7 and P8). Scale bars, 30 nm (**b**).

**Extended Data Fig. 5:**
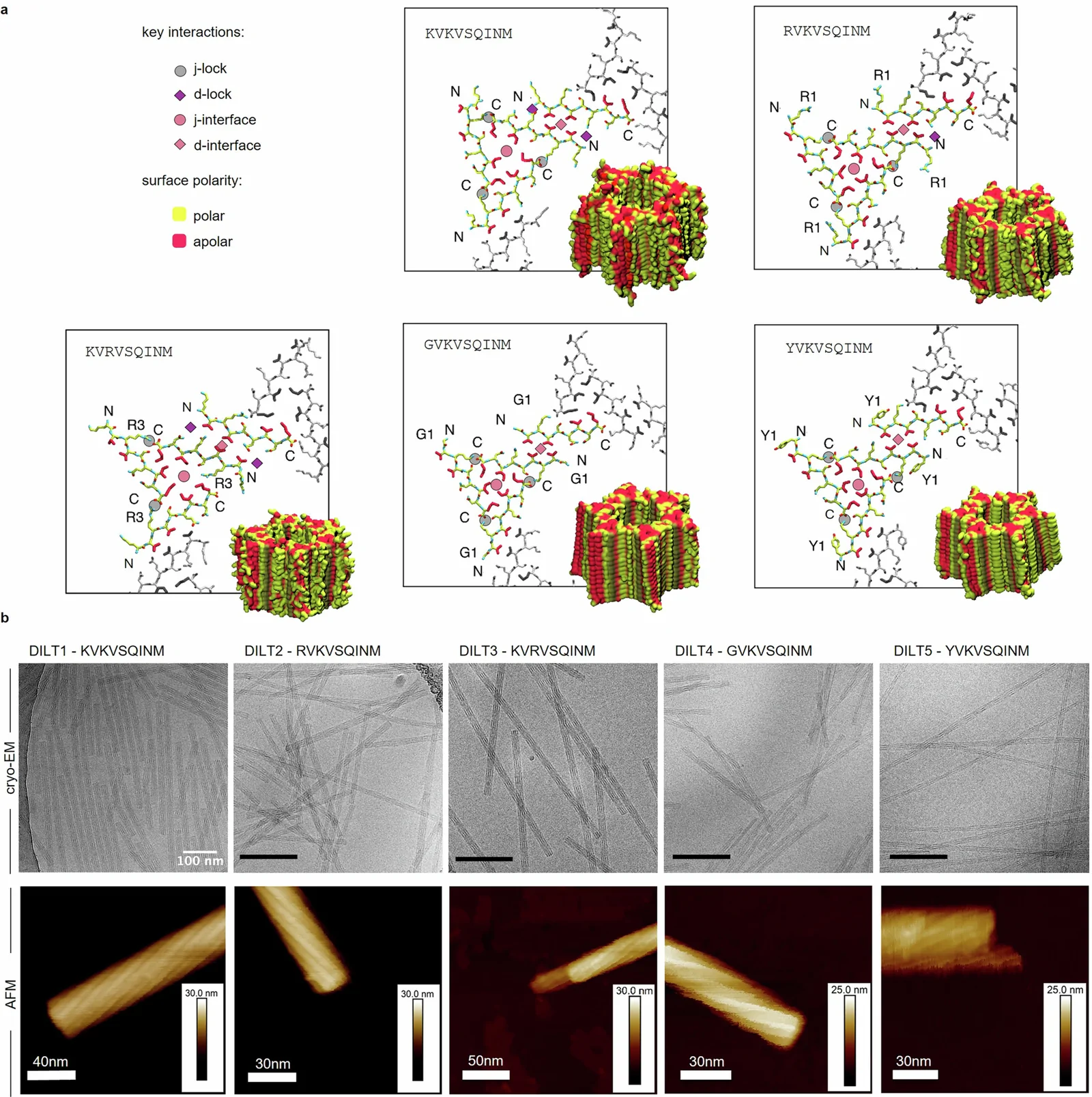
Molecular dynamics, cryo-EM and AFM analysis of honeycomb-forming DILT peptides. **a**, Final snapshots from 100 ns molecular dynamics simulations of 15-layer fibril fragments of DILT1 (KVKVSQINM), DILT2 (RVKVSQINM), DILT3 (KVRVSQINM), DILT4 (GVKVSQINM) and DILT5 (YVKVSQINM), showing assembly of the junction and cross-β motifs into hexagonal nanostructures. The interaction motifs are indicated by coloured circles and diamonds. Insets show the final three-dimensional structures of the 15-layer assemblies. **b**, Cryo-EM and AFM screening of DILT1–DILT5 (1 mg mL^−1^, 10 % v/v DMSO, 90 % v/v DPBS, room temperature), showing formation of multichannel fibrils with the characteristic grooved surface topology. Scale bars in cryo-EM micrographs, 100 nm.

To define the sequence constraints required for hexagonal lattice formation under the standard DILT assembly conditions, we systematically perturbed residues within the dimer, junction and inversion motifs. All variants were screened under matched concentration and buffer conditions, allowing direct comparison of their assembly behaviour within this defined regime. We evaluated the structural outcome by cryo-EM, analysis of the characteristic stripe pattern and molecular dynamics simulations. This perturbation analysis yields experimentally validated sequence–structure rules for hexagonal nanochannel assembly (Fig. 5a). Conservative substitution at the N-terminal X_1_ residue (DILT2) and variants carrying non-conservative substitutions at the N-terminus (DILT4 and DILT5) preserved hexagonal symmetry and stripe pattern organization, implying that this position is able to tolerate sequence variation without detectable loss of lattice organization. In molecular dynamics trajectories, the d-locks involving N8 and K1 are transient in DILT1 and can be replaced by the outwards projection of the K1 side-chain into the nanochannel, indicating that individual d-lock interactions contribute to stabilization but are not required for maintenance of global topology (Figs. 3 and 5a and Extended Data Fig. 3d). Consistent with this interpretation, non-conservative substitutions at position 1 (G in DILT4; Y in DILT5) abolish this d-lock interaction yet retain honeycomb architectures, identifying the N-terminus as a symmetry-tolerant site that modulates surface chemistry. Atomic force microscopy further reveals the grooved surface topology expected for multichannel fibrils and well-defined fibril boundaries, consistent with the presence of laterally expanded nanochannel architectures (Fig. 5b and Extended Data Fig. 5b).

**Extended Data Fig. 6:**
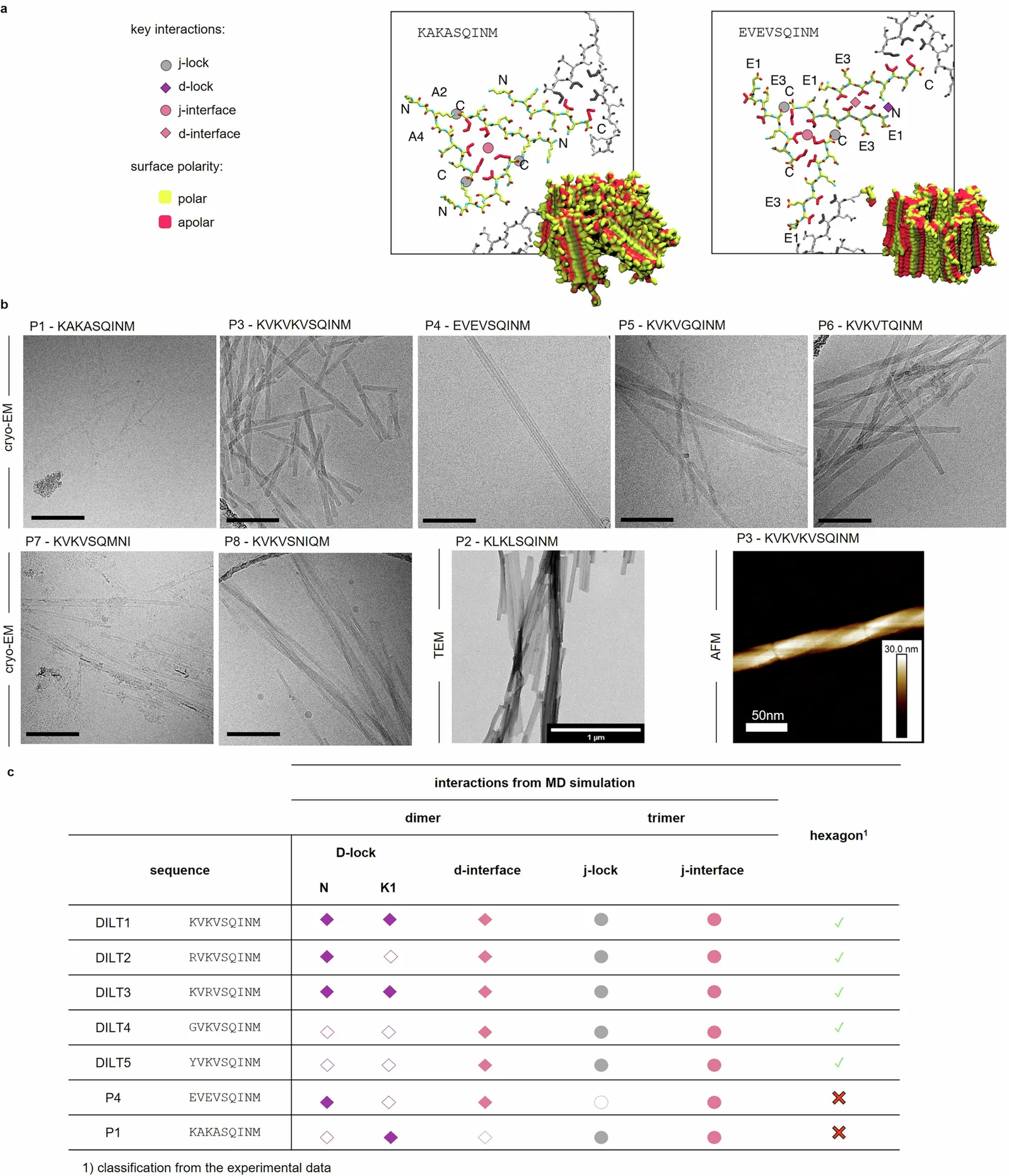
Molecular dynamics, cryo-EM, transmission electron microscopy (TEM) and AFM analysis of peptide variants P1–P8 showing absence of detectable hexagonal assembly under matched screening conditions and interactions from MD simulations. **a**, Final snapshots from 100 ns molecular dynamics simulations of 15-layer fibril fragments of selected peptide variants, illustrating that perturbation of essential interaction motifs does not support stable hexagonal nanochannel formation. The interaction motifs are indicated by coloured circles and diamonds. Insets show the final three-dimensional structures of the 15-layer assemblies. **b**, Cryo-EM, transmission electron microscopy (TEM) and AFM screening of peptide variants P1–P8 (1 mg mL^-1^ 10 % v/v DMSO, 90 % v/v DPBS, room temperature), showing loss of the characteristic striped fibril morphology and indicating absence of detectable hexagonal honeycomb assemblies. Scale bars in cryo-EM micrographs, 100 nm. **c**, Summary of the interactions in DILT1-5 peptides derived from the perturbation analysis, highlighting symmetry-tolerant and symmetry-critical positions for hexagonal lattice formation. P1 and P4 are listed for comparison.

In contrast, perturbation of the hydrophobic dimer interface did not yield detectable hexagonal assembly under these matched screening conditions. Alanine or leucine substitution (P1, A2/A4; P2, L2/L4, Supplementary Fig. 2) alters V2/V4-mediated packing and cross-β pairing, and results in loss of the stripe pattern under these conditions, indicating that the hydrophobic dimer interface is required for the formation of the ordered hexagonal lattice in the DILT assembly regime examined here. Moreover, extension of the amphiphilic dimer motif (P3, KVKVKVSQINM) yields fibrils lacking the stripe pattern, consistent with the loss of hexagonal lattice formation under the tested conditions. Charge inversion at the X_3_ position (P4, E1/E3) is expected to perturb junction stabilization and suppress lateral fusion, resulting in fibrils that likewise lack the characteristic intermittent stripe pattern. The inversion residue is positioned at the junction between the dimer-forming and trimer-forming segments and disrupts the otherwise regular amphiphilic alternation, consistent with a role in coupling the two interfaces geometrically, and participates in j-lock formation. Accordingly, mutations that alter this residue (P5, S5G; P6, S5T) produce fibrils with crossover but lacking stripe patterns, indicating loss of hexagonal organization. Similarly, perturbation of the trimeric QINM junction motif, including geometric reversal of hydrophobic (P7, M7/I9) or hydrophilic side chains (P8, N6/Q8), does not produce detectable honeycomb formation under the matched screening conditions and yields predominantly non-striped fibrils, underscoring the requirement for precise spatial positioning of residues within the trimeric interface (Fig. 5c and Extended Data Fig. 6a,b).

Together, these results define a hierarchical set of sequence constraints for hexagonal lattice formation under the matched assembly conditions used here (Extended Data Fig. 6c). The N-terminal position is symmetry tolerant and primarily modulates surface chemistry, whereas the hydrophobic dimer interface, the inversion residue and the trimeric junction are required to preserve the cooperative interactions that support cross-β-registry, lateral tiling and honeycomb topology in this assembly regime. These comparative data do not constitute an exhaustive phase diagram for each variant, and future studies may reveal additional morphologies under different buffer, ionic-strength or dilution conditions.

## Nanochannel hydration

**Extended Data Fig. 7:**
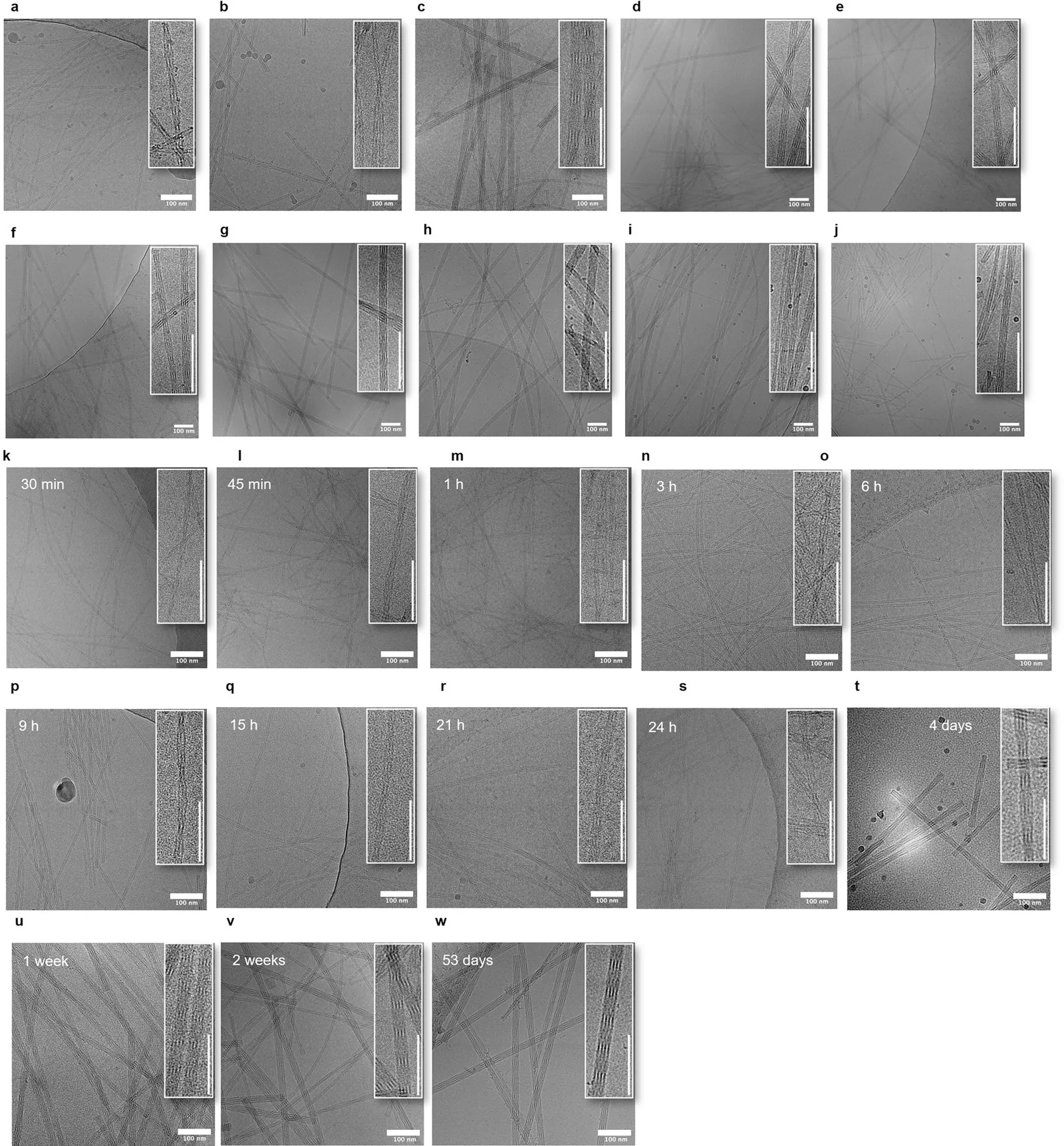
Fibril stability screening under different solvent, temperature and energy conditions, and kinetics of DILT1 fibril formation. **a**, Cryo-EM micrographs of fibrils assembled in 10 % v/v DMSO, 90 % v/v PBS (method I), **b**, pure PBS (method II) and **c**, in a diluted state (0.1 mg mL ^−1^) with 1 % v/v DMSO, 9 % v/v PBS, and 90 % v/v MilliQ H_2_O (method III). Cryo-EM micrographs of preformed fibrils after 15 min at **d**, 60 °C **e**, 85 °C and **f**, 95 °C and after 60 min at **g**, 60 °C, **h**, 85 °C and **i**, 95 °C (method VI). Fibrils are detected in all conditions. Above 85 ° and 60 min, the fibril’s curvature appears to increase (h and f). **j**, Cryo-EM micrograph of preformed fibrils after 15 min in a sonication bath (method VII), demonstrating that the fibril did not disassemble. Inset is a close-up of the fibrils. Scale bar = 100 nm. Cryo-EM micrographs of fibrils in the initial assembly stages (method IV) while shaking at 22 °C: **k**, 30 min, **l**, 45 min, **m**, 1 h, **n**, 3 h, **o**, 6 h, **p**, 9 h, **q**, 15 h, **r**, 21 h, **s**, 24 h. Inset are a close up of the fibrils. Cryo-EM micrographs of fibrils (method V) after **t**, 4 days, **u**, 1 week, **v**, 2 weeks, **w**, 53 days of incubation at 37 °C. Inset is a close-up of the fibrils. Scale bars, 100 nm.

**Extended Data Fig. 8:**
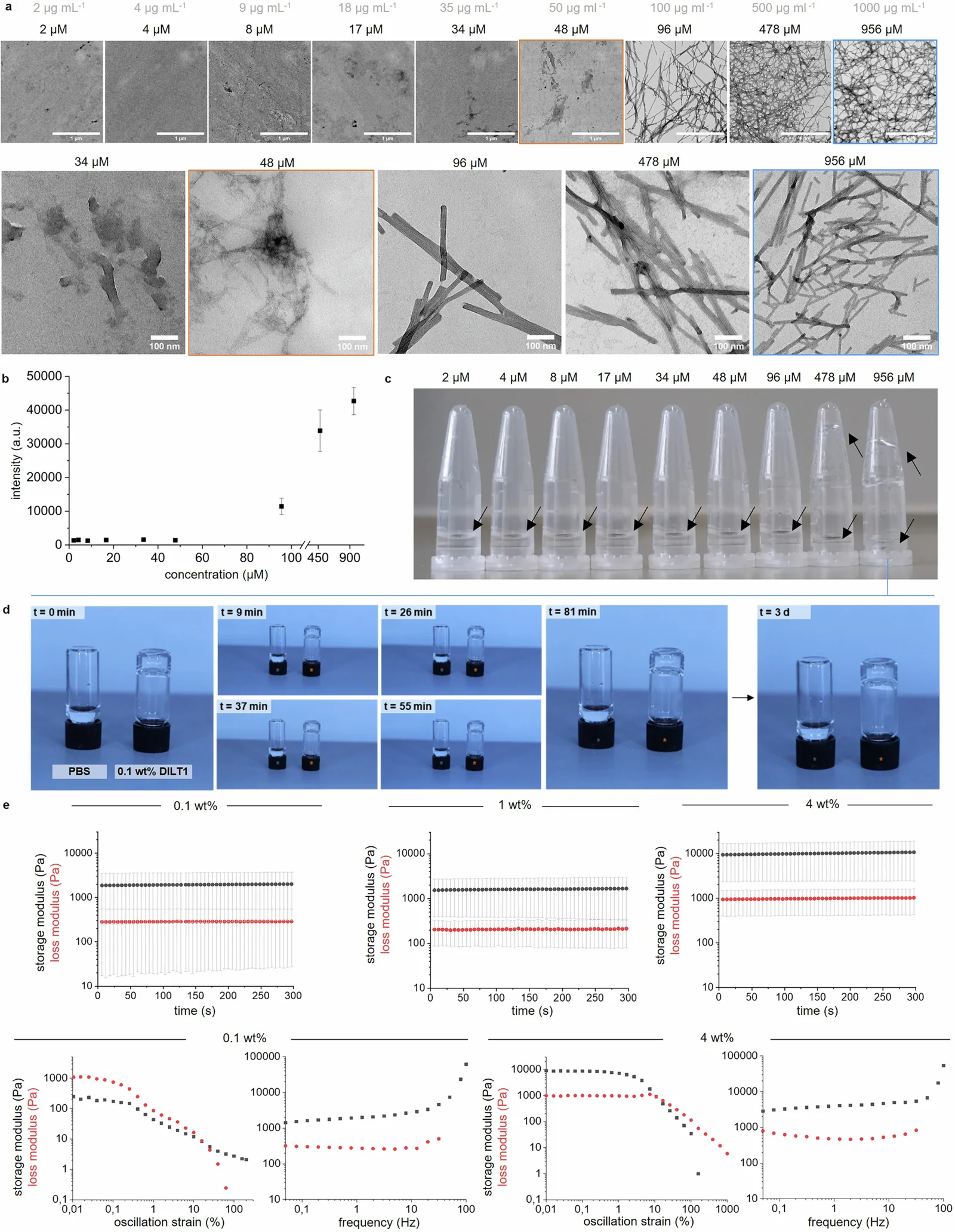
Characterisation of concentration-dependent self-assembly and gelation of DILT1. **a.** TEM of DILT1 under variation of concentration (2 µg mL^−1^-1.0 mg mL^−1^, 10 % v/v DMSO, 90 % v/v DPBS, overnight, room temperature). **b.** Critical aggregation concentration via proteostat® assay, technical replicates n = 2 (956 µM), n = 3 (2 µM – 478 µM) as mean with standard deviation. **c.** DILT1 examination of gelation behaviour via vial flip test. **d.** Macroscopic material DILT1 hydrogel. Stability examined via vial flip test over time. DPBS (left vial, ctrl, blue) and 0.1 wt% DILT hydrogel (right vial, orange) were inverted and left for incubation for 3 days at room temperature. **e,** Rheological characterisation of DILT1 mechanical properties under variation of concentration. Upper panel displays representative sections (~300 s, with indicative time stamps at approximately 6-second intervals) of oscillatory time sweeps of DILT1 0.1 wt% (prepared from predissolved DMSO peptide stock solution 10 mg ml^−1^ to DPBS, pH 7, N = 4), as well as 1 wt% and 4 wt% after gelation (prepared from peptide powder, DPBS, pH 7, N = 4 and N = 3 as mean with standard deviation, respectively). Lower panel displays oscillatory strain and frequency sweeps for 0.1 wt% (from predissolved DMSO peptide stock solution 10 mg ml^−1^ to DPBS, pH 7) and 4 wt% (from peptide powder, DPBS, pH 7) DILT1, respectively.

Under the tested aqueous buffer conditions, the self-assembly of these elements to form micrometre-long honeycomb fibrils is initiated within minutes. After overnight incubation, the formed honeycomb fibrils remain structurally intact in solution and these assemblies tolerate dilution, heating to 95 °C and sonication, indicating cooperative stabilization by the same cross-β- and junction interfaces that define the hexagonal nanochannel topology at the molecular scale (Extended Data Fig. 7). Fibrils are observed at concentrations above approximately 48 µM when incubated overnight, as shown by a concentration-dependent analysis of DILT1 by transmission electron microscopy (TEM) together with a fluorescence-based aggregation assay (Extended Data Fig. 8a,b). The DILT1 fibrils form self-supporting hydrogels, as demonstrated by vial inversion and mechanical property characterization, through reversible physical cross-links most likely between solvent-exposed peripheral interfaces (Extended Data Fig. 8c–e). Although the detailed pathway linking nanoscale integer tiling in DILT1 and interfibril interactions to macroscopic network formation remains to be resolved, these data link concentration-dependent fibril formation and network connectivity to macroscopic gelation.

**Fig. 6:**
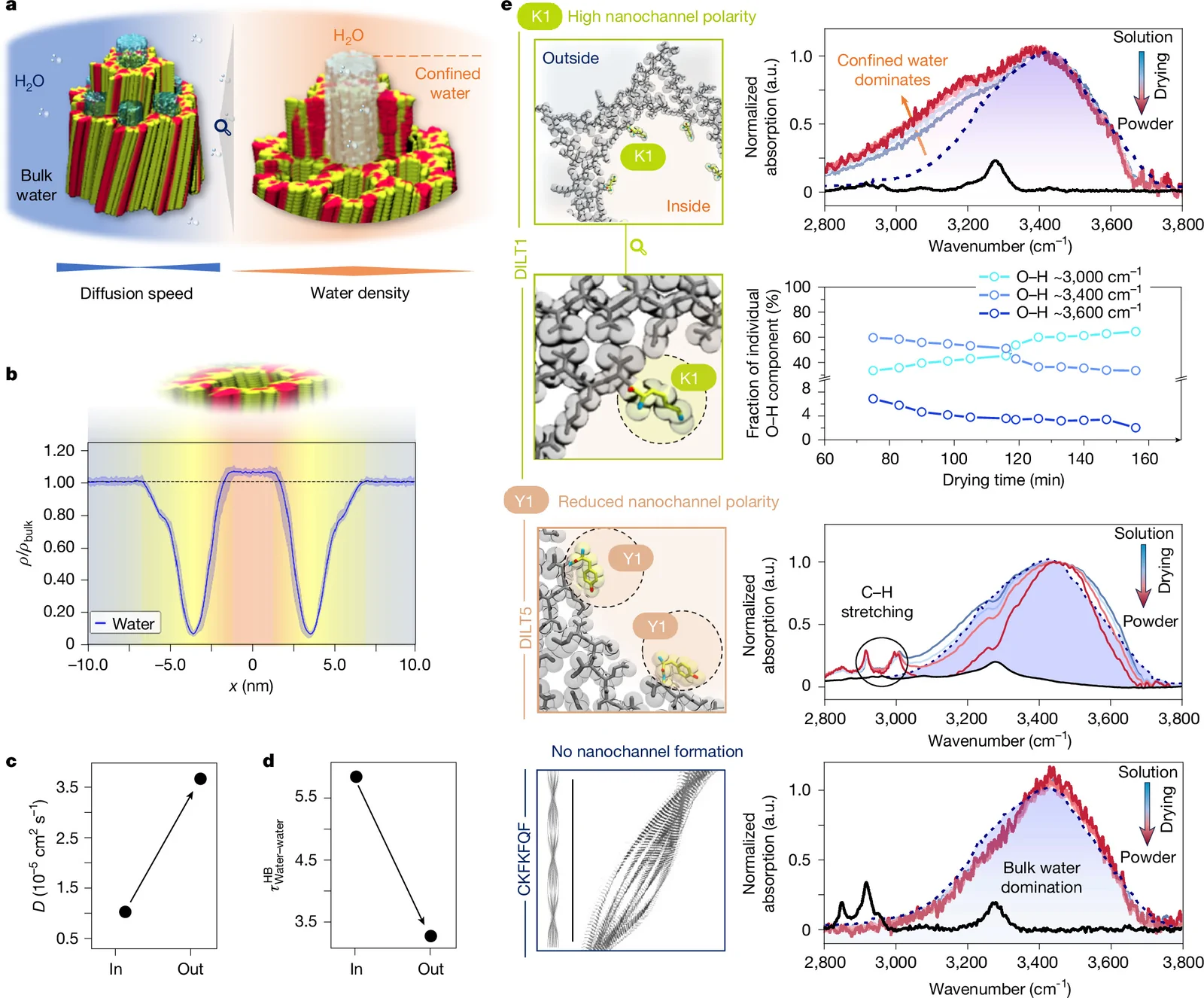
Topology-defined hydration in hexagonal nanochannel fibrils. **a**, Schematic of the honeycomb fibril with a continuous nanochannel containing confined water. Reduced diffusion and an axial density profile indicate hydration without interfacial drying. **b**, Radial water-density (*ρ*) normalized to the bulk value (*ρ*_bulk_) from molecular dynamics simulations of a DILT1 94-layer fibril showing complete wetting, elevated density within the pore and finite density at the peptide interface. **c**, Diffusion coefficients (*D*) for confined (in) and bulk (out) water demonstrating reduced mobility (DILT1). **d**, Water–water hydrogen-bond lifetimes ${\tau }_{\mathrm{Water}\mbox{--}\mathrm{water}}^{{\rm{HB}}}$ for confined (in) and bulk (out) water demonstrating prolonged hydrogen bonding inside the channel (DILT1). **e**, Tuning of topology-defined hydration by wildtype to X_1_ nanochannel surface polarity perturbations. Normalized FTIR spectra of hydrated assemblies during controlled dehydration. O–H vibrational absorption spectra of DILT1 (O–H band broadening) and DILT5 (O–H band narrowing) recorded at successive drying times, as well as the published control-peptide CKFKFQF^41,42^ (O–H band unaffected), which forms cross-β-assemblies without nanochannel topology (peptide nanofibril (PNF) scheme based on Protein Data Bank entry 8OKR^41^). Coloured traces represent spectra recorded at successive drying times, with the corresponding progression from nanofibril solution state to dry powder indicated by the colour bar; the individual spectra follow the same colour sequence. The black trace shows the fully dry peptide spectrum and the dashed trace shows bulk water for reference. The orange-shaded region marks the lower-wavenumber O–H stretching contribution associated with more strongly hydrogen-bonded and confined water, whereas the blue-shaded region marks the higher-wavenumber O–H contribution associated with bulk water. Drying evolution of individual O–H component fractions within the overall O–H stretching mode in DILT1 as a function of time (deconvolution of the 2,700–3,800 cm^−1^ region into one narrow N–H component near 3,300 cm^−1^ and three broad O–H components near 3,000 cm^−1^, 3,400 cm^−1^ and 3,600 cm^−1^) shows a preferential loss of more weakly associated water during early drying and relative enrichment of strongly hydrogen-bonded confined water at later drying stages.

**Extended Data Fig. 9:**
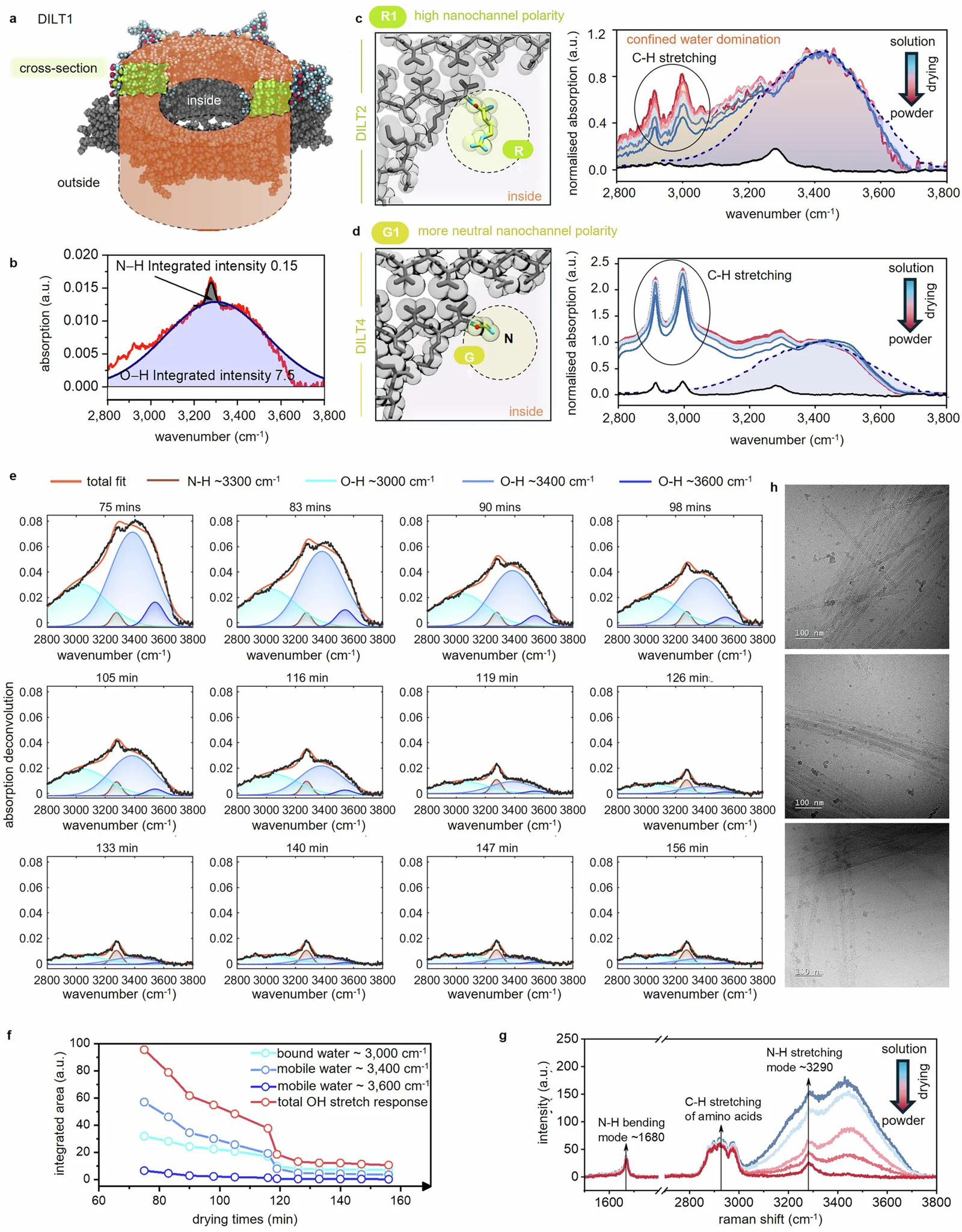
Molecular dynamics analysis and dehydration-dependent spectroscopy of nanochannel hydration. **a**, Cylindrical density profile of the principal species in solution from the simulation of a 94-layer DILT1 fibril. Water density is elevated inside the fibril compared with bulk solution. **b**, FTIR spectrum of DILT1 (see also main text Fig. 6e) recorded after drying for 116 min. The deconvolution-derived N–H/O–H integrated area ratio was used together with literature absorption cross-sections to obtain a semi-quantitative, model-dependent estimate of approximately 25 water molecules per peptide at the analysed drying stage. **c**,**d**, FTIR absorption spectra of the DILT2 and DILT4 samples at various drying times, corresponding to different water contents. Left: Schematic representation based on atomic model and MD simulation of DILT2 and DILT4, highlighting the nanochannel chemistry profile, respectively. C-H modes are assigned to residual DMSO solvent in the samples. Baseline-corrected spectra; right: Spectra normalized to the intensity at 3400 cm^−1^. **e**, Representative FTIR spectrum of DILT1 at the analysed drying stage. Spectra recorded during controlled dehydration were fitted over 2700–3800 cm^−1^ using one narrow N–H component near 3300 cm^−1^ and three broad O–H components centred near 3000, 3400 and 3600 cm^−1^. The ~3000 cm^−1^ component is assigned to more strongly hydrogen-bonded water, whereas the ~3400 and ~3600 cm^−1^ components are assigned to more weakly hydrogen-bonded or mobile water. The deconvolution-derived N–H/O–H integrated area ratio was used together with literature absorption cross-sections to obtain a semi-quantitative, model-dependent estimate of approximately 25 water molecules per peptide at the analysed drying stage. **f**, Integrated area of different O–H components at the analysed drying stage. **g**, Drying-dependent Raman spectra of DILT1 showing no detectable frequency shifts of peptide C–H or N–H modes between hydrated and powder-like states within the sensitivity of the measurement. **h**, Cryo-EM of semi-dry DILT1. DILT1 (1 mg ml^−1^, DPBS, 110 min of drying under the same dehydration protocol used for the FTIR measurements) displays fibrillar structures with features consistent with the characteristic hexagonal honeycomb fibril fingerprint stripe pattern.

The fibrillar lattice contains a continuous solvent-accessible nanochannel whose physicochemical properties are modulated by the amino acid exposed to the pore interior. Molecular dynamics simulations reveal a water-filled channel with elevated water density relative to bulk solvent and reduced diffusional mobility along the radial axis (Fig. 6a–d, Extended Data Fig. 9a and Supplementary Fig. 3). To probe pore hydration experimentally, Fourier-transform infrared (FTIR) spectroscopy was performed during controlled dehydration at defined water contents (Fig. 6e and Extended Data Fig. 9b–g). Normalized FTIR spectra after subtraction of the corresponding fully dry peptide fibril spectrum highlight the water-related O–H stretching response during dehydration. In DILT1, progressive dehydration leads to a broadened and redshifted normalized O–H stretching envelope, where a low-wavenumber component distinct from bulk liquid water becomes increasingly pronounced (Fig. 6e), consistent with an increasingly heterogeneous and more strongly hydrogen-bonded water population during drying. Because the O–H stretching region overlaps with peptide N–H contributions and contains multiple water populations, we analysed the dehydration series by deconvoluting the 2,700–3,800 cm^−1^ region into one narrow N–H component near 3,300 cm^−1^ and three broad O–H components centred at approximately 3,000 cm^−1^, 3,400 cm^−1^ and 3,600 cm^−1^ (refs. ^39,40^; Extended Data Fig. 9e,f).

During DILT1 drying, the total integrated O–H response decreases continuously, whereas the higher-wavenumber O–H components (approximately 3,400 cm^−1^ and approximately 3,600 cm^−1^) assigned to more weakly hydrogen-bonded or mobile (bulk-like) water decay more rapidly than the low-wavenumber component near 3,000 cm^−1^ assigned to strongly hydrogen-bonded and more confined water. Consequently, the relative contribution of the strongly hydrogen-bonded water population increases during drying and dominates at later time points before being progressively depleted. These data support preferential removal of more weakly associated external or mobile water molecules, followed by retention and gradual loss of more strongly hydrogen-bonded confined water, rather than abrupt channel emptying. By contrast, the cross-β-control-peptide CKFKFQF^41,42^, which forms β-sheets but lacks a nanochannel topology, shows a progressive reduction in the overall O–H stretching response under dry nitrogen while the bandwidth remains essentially unchanged, consistent with uniform water loss rather than confinement-dependent dehydration.

For DILT1, a semi-quantitative estimate based on deconvolution-derived N–H/O–H integrated band areas and literature infrared absorption cross-sections, as described in Methods, yields approximately 25 confined water molecules per peptide at around 120 min in the drying stage when the relative fraction of strongly bound water reaches a plateau (Fig. 6e and Extended Data Fig. 9f), consistent with a fully filled nanochannel. This estimate is inherently approximate and model dependent as it relies on environment-sensitive absorption coefficients, separation of overlapping N–H and O–H bands, and assumptions about the number of peptide N–H oscillators contributing to the spectrum.

For DILT2, DILT4 (Extended Data Fig. 9c,d) and DILT5 (Fig. 6e), the presence of confined water is likewise inferred based on the observations made for DILT1. Importantly, the O–H stretching envelopes (Fig. 6e and Extended Data Fig. 9e,f) indicate that sequence variation at the pore-lining position can tune the local hydrogen-bonding state of the confined water in both directions: K1 and R1 substitutions shift the band to a lower wavenumber, consistent with enhanced hydrogen bonding, whereas G1 shows no discernible shift, suggesting hydrogen bonding comparable to that of bulk water. For DILT5 containing the more lipophilic Y1 residue, we do not observe the characteristic dehydration profile in the O–H stretching region, suggesting that the water environment in DILT5 differs from that in the other samples, which may reflect differences in peptide–water interactions arising from variations in local electrostatic environments. DILT1 (K1), DILT2 (R1), DILT4 (G1) and DILT5 (Y1), therefore, illustrate sequence-controlled modulation of nanochannel polarity while preserving the hexagonal lattice. In particular, replacing K1 with R1 (DILT2; Extended Data Fig. 9c) preserves a highly polar channel environment, whereas replacing it with G1 (DILT4; Extended Data Fig. 9d) or Y1 (DILT5; Fig. 6e) reduces the effective polarity of the pore surface. We next examined whether dehydration detectably alters the peptide architecture. Raman spectra recorded during drying show no detectable frequency shifts of the peptide C–H or N–H modes between hydrated and powder-like states, although the overall intensities change as water is removed (Extended Data Fig. 9g). The FTIR spectra likewise show that the peptide N–H stretch near 3,300 cm^−1^ remains essentially unchanged in frequency during dehydration. In addition, electron microscopy of samples dried for around 120 min under the same conditions used for FTIR measurements retains fibrillar structures with features consistent with the characteristic hexagonal fibril fingerprint (Extended Data Fig. 9h). These observations indicate that dehydration does not cause detectable collapse of the peptide architecture within the sensitivity of the vibrational and electron-microscopy measurements, although a full diffraction-based comparison of hydrated and dehydrated states will be required to quantify changes in crystallinity. Together, molecular dynamics simulations and vibrational spectroscopy show that the pores are not empty geometric cavities, but solvent-accessible nanochannels whose confined water can be tuned between more strongly and more weakly hydrogen-bonded states by sequence variation at a single pore-lining residue.

We demonstrate that a minimal linear peptide sequence can encode a symmetry-defined supramolecular architecture through a hierarchy of complementary interaction motifs. By integrating a cross-β-dimer interface, a trimeric junction, an inversion element and locking interactions within a nine-residue scaffold, amphiphilic peptides assemble into laterally expandable honeycomb lattices containing continuous hexagonal nanochannels. This architecture emerges directly from sequence-encoded geometry rather than from pre-organized cyclic scaffolds, folded protein domains or metal coordination, showing that programmable higher-order organization can arise from minimal β-sheet building blocks. Systematic sequence perturbation identifies symmetry-critical and symmetry-tolerant positions, establishing design rules for laterally expandable β-sheet multichannel nanofibrils.

Unlike conventional amyloid fibrils, which predominantly elongate through repetitive stacking and achieve macroscopic cohesion through polymorphic entanglement, the assemblies reported here expand laterally by integer tiling of a conserved hexagonal unit. This mechanism generates multichannel fibrils with conserved pore geometry across sequence variants within the set experimental conditions and links molecular sequence to mesoscale architecture. Although the present data do not resolve the exact growth pathway, the hexagonal honeycomb nanochannels probably form through a nucleation-and-growth process in which initial assembly of a hexagonal unit is coupled to subsequent lateral tiling and axial propagation. Future studies will be needed to define the precise sequence and relative contributions of these growth steps, involving the role of buffer ions, concentrations, kinetic and thermodynamic factors, as well as environmental parameters. The resulting pores are solvent accessible and show sequence-dependent hydration, indicating that molecular sequence can tune not only the supramolecular topology but also the physicochemical environment within confined spaces. The same interfaces that define nanoscale symmetry also support reversible interfibrillar association and hydrogel formation, although the detailed pathway from integer tiling to macroscopic network formation remains unresolved. More broadly, these results establish short linear β-sheet peptides as modular units for programmable, symmetry-defined lateral growth, opening opportunities for multichannel biomolecular materials with tunable confined solvation and transport-related properties.

## Methods

### Materials

Chemicals were purchased from commercial suppliers (Thermo Scientific, Merck KGaA, VWR and Honeywell). Solvents and reagents used for peptide synthesis were obtained in peptide grade. Solvents used for peptide purification were of high-performance liquid chromatography (HPLC) grade; dimethyl sulfoxide (DMSO) was BioUltra grade for molecular biology (≥99.5%, Sigma Aldrich); and water was obtained from a Millipore purification system. Dulbecco’s phosphate buffered saline (DPBS; 1x, without CaCl_2_ and MgCl_2_, composition: KCl 0.2 g l ^−1^, KH_2_PO_4_ 0.2 g l ^−1^, NaCl 8.0 g l ^−1^, Na_2_HPO_4_ (anhydrous) 1.15 g l ^−1^) was purchased from Sigma Aldrich (DPBS) or from Thermo Fisher Scientific (Dulbecco’s balanced salt solution) with minor variations in inorganic salt water content. DILT1 was in part purchased from GenScript Biotech with a purity of ≥98%. DILT2-5 and peptides P1 and P3–P8 were purchased from GenScript Biotech with a purity of ≥98%.

### Solid-phase peptide synthesis of DILT1 (KVKVSQINM) and P2 (KLKLSQINM)

For peptide synthesis, the fluorenylmethyloxycarbonyl (Fmoc) solid-phase peptide synthesis strategy was applied from the C-terminus to the N-terminus using *N*,*N*′-diisopropylcarbodiimide (DIC) and ethyl cyano(hydroxyimino)acetate (Oxyma) on an automated microwave-assisted peptide synthesizer (CEM, Liberty Blue). The peptides were synthesized onto pre-swollen Fmoc-Met-Wang resin (0.1 mmol, 0.147 g, substitution 0.68 mmol g^−1^, 100–200 mesh, pre-swollen in dimethylformamide (DMF; 2–3 ml) on a shaker at room temperature) by sequential coupling of activated Nα-Fmoc-amino acid in DMF (2.5 ml, 0.2 M) in the presence of DIC (1 ml, 0.5 M) and Oxyma (0.5 ml, 1.0 M) in DMF via microwave-assisted reaction at 75 °C (170 W) for 15 s and 90 °C (30 W) for 110 s followed by multiple washing of the resin (DMF, 2 × 3 ml, 1 × 4 ml). Sequentially, for DILT1 Fmoc-Asn(Trt)-OH, Fmoc-Ile-OH, Fmoc-Gln(Trt)-OH, Fmoc-Ser(tBu)-OH, Fmoc-Val-OH, Fmoc-Lys(Boc)-OH, Fmoc-Val-OH and Fmoc-Lys(Boc)-OH were coupled to the resin via cycling coupling with each amino acid introduced in one sequential coupling cycle, washing, and Nα-Fmoc-deprotection steps. For P2, Fmoc-Leu-OH was used instead of Fmoc-Val-OH. Nα-Fmoc deprotection was conducted by application of piperidine (3 ml, 20% v/v in DMF) under heating (75 °C, 155 W, 15 s, followed by 90 °C, 30 W, 50 s), followed by washing with DMF (2 × 2 ml, 1 × 3 ml). The synthesized peptides on resin were kept in DMF at 4 °C until further use. Subsequently, the peptides were cleaved from the resin using a cleavage cocktail (10 ml) comprising trifluoroacetic acid (TFA) (95% v/v), Milli-Q water (2.5% v/v) and triisopropylsilane (TIPS) (2.5% v/v). After 2 h incubation on a shaker at room temperature, the peptides were precipitated with diethyl ether pre-cooled to 4 °C (40 ml) and centrifuged at 4,000 rpm for 16 min (×2, 0 °C). The resultant crude products were dried overnight at room temperature. The crude products were purified by preparative reverse-phase HPLC using a 0.1% TFA water–acetonitrile mixture as eluant and lyophilized to yield the peptide as a white amorphous powder. The purified peptides were characterized by matrix-assisted laser desorption/ionization time-of-flight mass spectrometry (MALDI-TOF MS). The lyophilized solids were stored at −20 °C until use.

MALDI-TOF MS (DILT1): *m*/*z*_calc_ = 1,045.60, *m*/*z*_found_: 1,046 [M + H]^+^, 1,062 [M + O + H]^+^, 1,068 [M+Na]^+^, 1,084 [M + K]^+^, 1,100, 1,106.

MALDI-TOF MS (P2): *m*/*z*_calc_ = 1,073.63, *m*/*z*_found_: 1,074 [M + H]^+^, 1,090 [M + O + H]^+^, 1,096 [M+Na]^+^, 1,112 [M + K]^+^, 1,128, 1,134, 1,150, 568.

### High-performance liquid chromatography

The synthesized crude peptide was purified via preparative HPLC of the dissolved and filtered (0.22 µm) precipitate (Shimadzu system). Multiple manual injections (2× or 3 × 8 mL;) of the peptide crude solution (*c* = 1.3 mg ml^−1^, water with 0.1% TFA (v/v) and 5% acetonitrile (ACN) (v/v)) were performed. Separation was performed by a preparative reverse-phase HPLC column (Phenomenex 5 µm C18, 150 × 30 mm) using a 0.1% TFA water–ACN mixture as eluant (flow 25 ml min^−1^), the applied gradient was as follows: (1) 0.01 min: 5% ACN, 0.1% TFA (A) with 95% 0.1% TFA in water (B); (2) 5 min: 5% A with 95% B; (3) 17 min: 70% A with 30% B; (4) 19 min: 100% A with 0% B; (5) 24 min: 100% A with 0% B; (6) 28 min: 5% A with 95% B; (7) 30 min: 5% A with 95% B. Chromatography was monitored with an ultraviolet absorption detector at 214 nm. Alternatively, the gradient can be applied as follows (for example, P2); (1) 0.01 min: 0% ACN, 0.1% TFA (A) with 100% 0.1% TFA in water (B); (2) 5 min: 0% A with 100% B; (3) 15 min: 40% A with 60% B; (4) 17 min: 100% A with 0% B; (5) 19 min: 100% A with 0% B; (6) 21 min: 5% A with 95% B; (7) 24 min: 5% A with 95% B. Data were evaluated in LabSolutions software.

### MALDI mass spectrometry

Peptide mass spectra were recorded in positive ion reflector mode on a Bruker rapifleX MALDI-TOF/TOF mass spectrometer (Bruker Daltonik, scanning smartbeam 3D 10-kHz neodymium-doped yttrium aluminium garnet (Nd:YAG) laser, wavelength of 355 nm, and 10-bit 5-GHz digitizer, acceleration voltage 20 kV) using α-cyano-4-hydroxycinnamic acid (HCCA) as the matrix. Samples were measured with random walk ionization across the sample spot, and usually 8,000 shots were averaged per spectrum. The sample was prepared by mixing an acidic aqueous analyte solution (0.1% TFA) in equal amounts with the supernatant of a saturated HCCA solution acetonitrile/water (30:70 + 0.1% TFA). Subsequently, this mixture (1 µl) was applied to a stainless steel target and allowed to dry before measurement. Calibration was conducted with sodium adducts of polyethylene glycol in a mass range of 500–3,000 Da. Data were evaluated with mMass software.

### MSMS sequencing

The sequence analysis was carried out on a rapifleX MALDI-TOF/TOF mass spectrometer from Bruker Daltonik. The instrument is equipped with a scanning smartbeam 3D 10-kHz Nd:YAG laser at a wavelength of 355 nm and a 10-bit 5-GHz digitizer. The acceleration voltage was set to 20 kV, and the mass spectra were recorded in positive ion MSMS mode. The protonated molecular ion of the peptide at 1,046 Da was selected as precursor with an isolation window of +6 Da and −6 Da. Basic instrument calibration was done with the Bruker peptide mix and the Bruker protein calibration standard I and II in a mass range up to 70 kDa. Before fragment ion analysis, the instrument was manually recalibrated with the exact mass of the precursor ion at 1,046.5418 *m*/*z*. Samples were measured with random walk ionization across the sample spot. The laser power was adjusted to provide significant signal intensity for b- and y-series fragments, and 2,000 shots were averaged per spectrum. Sample preparation was done by mixing a 50 mg ml^−1^ solution of super-DHB matrix in acetonitrile/water (1:1 + 0.1% TFA) with equal amounts of the peptide solution (10 mg ml^−1^) in acetonitrile/water (1:1 + 0.1% TFA). Finally, 1 µl of the mixture was applied to a stainless steel target and allowed to dry before measurement. The mass spectrometer was controlled by the software Bruker flexControl (Version 4.0) and data analysis was done by Bruker flexAnalysis (Version 4.0).

### Peptide nanofibril formation

Nanofibril formation was induced by introducing the pre-dissolved peptide stock solution (10 mg ml^−1^, DMSO) into DPBS (pH 7.4), yielding a 1 mg ml^−1^ solution. After brief vortexing, the samples were incubated on a shaker (500 rpm, room temperature) overnight. Further incubation was conducted as indicated without shaking at room temperature or at 37 °C for the indicated periods.

### Peptide nanofibril electron microscopy screening

#### Cryo-EM

For cryo-EM micrograph screening of DILT2–DILT5 and peptides P1 and P3–P8, samples were incubated as described above (1 mg ml^−1^) overnight (500 rpm, 25 °C), subsequently kept at room temperature without shaking and plunge-frozen after 4 days. Holey-carbon-coated grids (400 mesh C-flat 1.2/1.3) were glow-discharged (PELCO easiGlow glow discharge cleaning system; TED PELLA), 3 µl sample was applied, blotted for 8 s and plunge-frozen in liquid ethane using an Automatic Plunge Freezer EM GP2 (Leica Microsystems) for each sample. Grids were screened on a JEM-2100 transmission electron microscope (Jeol) at 200 kV using a TVIPS F416 camera.

#### Transmission electron microscopy

P2 (L2/L4) was imaged after overnight incubation via TEM as described previously^42,43^. The pre-incubated sample (5 μl, 1 mg ml^−1^) was placed on a copper grid coated with formvar layer (etched with oxygen plasma). Following a short incubation, excess liquid was removed with filter paper, and the grids were stained with uranyl acetate solution (4% w/v, 2 min) and washed with water. Measurements were performed on a Jeol 1400 electron microscope with 120 kV acceleration voltage.

### Cryo-EM sample preparation and data acquisition

For DILT1 analyses, samples were incubated overnight (500 rpm, 22 °C), and further sample incubation was conducted without shaking at 37 °C for the indicated periods. For DILT2 (2-2-2) and DILT3 (2-2-2) cryo-EM reconstruction analyses, samples were incubated overnight (500 rpm, 25 °C), subsequently kept at 37 °C without shaking, and plunge-frozen after 7 days and 1 day, respectively. In case of DILT1 and DILT3, a 4 µl aliquot of the DILT1 fibril sample (from the indicated incubation time points) was applied on a Quantifoil grid (Q R2/2, 400 mesh) that was glow-discharged in a 6:1 oxygen/hydrogen plasma (Diener Nano, Diener Electronic) for 30 s shortly before. The excess solution was blotted for 4 s at 4 °C and >80 % humidity. The specimen was cryo-plunged in liquid ethane using a Vitrobot Mark V (Thermo Fisher Scientific). Subsequent imaging was done using a Titan Krios G4 transmission electron microscope (Thermo Fisher Scientific) at 300 kV, equipped with a Gatan GIF continuum spectrometer. Micrographs and videos were acquired on a 6,912 × 6,912-pixel K3 Gatan direct electron-detection camera with the spectrometer operated in imaging mode using a slit width of 20 eV. For data acquisition, the K3 camera was operated in super resolution and correlated double sampling mode. Extended Data Table 1 lists the data acquisition parameters.

For DILT2 (4-4-4) and DILT3 (3-3-3) cryo-EM reconstruction analyses, samples were incubated overnight (500 rpm, 25 °C), subsequently kept at room temperature without shaking, and plunge-frozen after 15 days for DILT2 and after 8 days for DILT3. For each, 3 µl of sample was applied to a glow-discharged (PELCO easiGlow glow discharge cleaning system; TED PELLA) holey-carbon-coated grid (400 mesh C-flat 1.2/1.3). After blotting with filter paper for 8 s and plunge-freezing in liquid ethane using an Automatic Plunge Freezer EM GP2 (Leica Microsystems), the grids were screened on a JEM-2100 transmission electron microscope (Jeol) at 200 kV, equipped with a TVIPS F416 camera. The dataset for reconstruction was recorded on a Krios G4 transmission electron microscope (Thermo Fisher Scientific) with a Falcon4i (Thermo Fisher Scientific) direct electron detector and a Selectris X energy filter (Thermo Fisher Scientific) operated with a 10-eV slit width. Extended Data Table 1 lists the data acquisition parameters.

### Helical reconstruction

In the case of DILT1 fibrils, MotionCor2^44^ was used for movie correction. Contrast transfer function (CTF) for all micrographs was performed using CTFFIND^45^. Further image processing and 3D helical reconstructions were performed with RELION-4.0^46,47^ on the 1-1-1 (hexagon fibril) and 3-3-3 (honeycomb fibril) morphologies. For the 3-3-3 morphology, micrographs containing ice or lacking the 4.8-Å-resolution information were excluded. The 3-3-3 morphology filaments were manually picked. As an initial model for the refinement, a featureless cylinder was used with a 60-Å low-pass filter and *C*_1_ symmetry. From the 3D auto-refinement, a *C*_6_ symmetry was observed and imposed, reaching a 4.3-Å-resolution volume with clearly visible β-sheets. To further improve resolution, the full dataset was re-processed via 3D classification and further 3D auto-refine to reach the final 3 Å volume. The volume was then post-processed with a soft-edge mask and sharpened with a B-factor of −50. All 3D classifications were carried out on the central 10% of the reconstruction. For the 1-1-1 morphology, a similar approach was performed. Details of particle extraction and helical reconstruction are reported in Extended Data Table 1.

In the case of the 2-2-2 DILT2 fibrils, CTF for all single images, acquired with drift correction in SerialEM, was performed using CTFFIND^45^. Further image processing and 3D helical reconstructions were performed with RELION 5.0^46^. The 2-2-2 morphology filaments were manually picked. As an initial model for the refinement, a simulated 2-2-2 hexagonal morphology was used. From the 3D auto-refinement, a *C*_3_ symmetry was imposed. The volume was then post-processed with a soft-edge mask and sharpened with a B-factor of −50. All 3D analyses were carried out on the central 10% of the reconstruction.

In the case of DILT2 (4-4-4), helical reconstruction was performed in RELION 5.0^46^. Raw video frames were aligned and corrected for beam-induced motion using RELION’s own implementation of MotionCorr2^44^, followed by CTF estimation using CTFFIND 4.1^45^. Fibrils displaying the 4-4-4 morphology were manually selected from the micrographs. Binned segments were extracted and subjected to reference-free two-dimensional classification until a homogeneous set of particles was obtained. Selected segments were extracted for 3D refinement without binning for 3D reconstruction. Helical refinement was performed using a low-passed cylinder and helical twist estimated from the raw micrographs by manual measurement in Fiji^48^, and a helical rise of 4.75 Å without the imposition of rotational symmetry. For the purpose of map improvement, rotational symmetries *C*_2_, *C*_3_ and *C*_6_ were imposed in the subsequent refinement, of which *C*_3_ symmetry yielded the best quality map as validated by visual inspection. Furthermore, the local search for helical parameters was turned on to optimize the twist and rise of the fibril. After the initial reconstructions, 3D classification and selection of the refined particle stack with progressive increase of the Tau fudge factor were carried out to remedy residual heterogeneity. The resulting particles were subjected to Bayesian polishing. The polished, final stack underwent two cycles of CTF refinement and reconstruction until no further improvement of the reconstructed map was noticeable. The final map was masked with a soft-edged mask and post-processed, yielding a map with a nominal gold-standard resolution of 1.9 Å.

In the case of DILT3 (3-3-3), image processing was performed in cryoSPARC 4.7.1^49^. Raw video frames were aligned and corrected for beam-induced motion using cryoSPARC’s internal patch motion correction. CTF parameter estimation was performed by patch CTF estimation. Exposures were manually curated to exclude micrographs with thick ice and fibril overcrowding. Filament segments were automatically picked using the filament tracer, and segments were extracted with binning for initial classification. Reference-free two-dimensional classification was used to separate segments belonging to different fibril morphologies. Particles corresponding to the 3-3-3 morphology were re-extracted without binning. The resulting stack was subjected to multiple rounds of two-dimensional classification to remove low-quality particles. An initial 3D helical reconstruction was performed using a featureless cylinder without the imposition of helical symmetry. The obtained map was used to bootstrap a 3D helical refinement with enforcement of helical symmetry parameters as obtained by measurement in Fiji^48^. Three-dimensional classification without particle alignment was used to resolve structural heterogeneity. Classes displaying clear separation of fibril layers along the helical axis were selected, and helical symmetry search was enabled during subsequent reconstructions. The homogeneous particle stack was subjected to reference beam motion correction in cryoSPARC and subsequent local and global CTF refinements. Several rounds of local CTF refinement and helical refinement were performed until no further improvement of the 3D map was observable. The final particle stack was symmetry expanded, and a final local refinement with recentring was performed. The resulting half-maps and reconstruction mask were imported into RELION 5.0^46^ where masking and post-processing were performed, yielding a final map with a gold-standard resolution of 1.78 Å. Details of particle extraction and helical reconstruction are reported in Extended Data Table 1.

### Atomic model building and refinement

In the case of DILT1, the atomic model for morphology 3-3-3 was built de novo using the program Coot^50^. Given the large number of chains present in the structure, model building was performed stepwise. The fundamental chains of the fibril are termed I.II.III–IV.V.VI–VII.VIII–IX.X (Extended Data Fig. 2g). Initially three chains (I-II-III) were constructed in Chimera^51^ by applying the standard β-parallel parameters ((*Φ*, *Ψ*) = (–135°, 135°)). These were manually aligned to the junction motif in the 3D volume. Next, to limit atomic clashes in the fibril axis, three layers of such chains were constructed. The three-layer system was refined further in Coot^50^ and Chimera^51^. An iterative optimization process was performed to achieve optimal fitting. Next, the structure was further extended to take into consideration the cross-β-motif. Once the I-II-III chains were optimized, they were imposed on the remaining outer electron density. MolProbity^52^, the comprehensive validation tool in Phenix^53^, was used to produce a validation of the atomic model to assess atomic clashes, rotamer and Ramachandran outliers, and model geometry. The structural statistics for refinement and model building are listed in Extended Data Table 1 and refer to the deposited atomic model (18 chains and 3 layers).

In the case of 2-2-2 DILT2 fibrils, the starting model for refinement was the atomic model of DILT1, and the applied rotational symmetry was *C*_3_. The structural statistics for refinement and model building are listed in Extended Data Table 1 and refer to the deposited atomic models.

In the case of DILT3 fibrils, an initial atomic model was generated de novo using ModelAngelo^54^. An iterative model refinement process was used in which an asymmetric unit of a single layer of the *C*_6_-symmetrical DILT3 fibril cross-sections was refined in WinCoot 1.1.18^49^. Phenix 2.0.5936^53^ was used to evaluate model geometry and clash score. ChimeraX 1.10^55^ was used to obtain a helical assembly of three layers, which was again validated. The three-layer model was further manually refined in WinCoot 1.1.18^49^ using regularization with Ramachandran, torsion and planar peptide restraints, followed by a subsequent real-space refinement. ISOLDE^56^ was used to improve the clash score. After each model refinement cycle, the contact sites between asymmetric units were checked for steric clashes by symmetry expansion to the original fibril symmetry and renewed validation in Phenix. Final images were produced using Fiji^48^ and ChimeraX^55^.

In the case of DILT2 fibrils, the same procedure was used as for DILT3 fibrils, with the exception of the starting model and the applied symmetry. The starting model for refinement of DILT2 was the atomic model of DILT3, and the applied rotational symmetry was *C*_3_. The structural statistics for refinement and model building are listed in Extended Data Table 1 and refer to the deposited atomic models.

### Polymorphism analysis

The CTF-corrected micrographs were used for the polymorphism analysis. A subset of 100 micrographs was randomly generated with the RELION ‘Subset Selection’ function. Each of these micrographs was analysed, and the different morphologies were manually picked and extracted using a box size of 350 pixels, nanochannel diameter of 220 Å, number of asymmetric units 7 and rise of 4.7 Å. The number of extracted particles was used to quantify the different morphologies. The polymorphism prediction was performed using Chimera^51^, RELION-4.0^47^ and Fiji^48^. Three layers of the atomic model of the central hexagon obtained from the 3-3-3 morphology were copied and shifted manually to create plausible hexagonal patterns. In Chimera^51^, the atomic model was converted into a volume with a 3-Å resolution (molmap #0 3) and saved as an .mrc file. The file was then processed with RELION-4.0^47^ to create a prolonged volume. Specifically, the file was converted into a file with a box size of 1,750 pixels, a nanochannel diameter of 250 Å, a rise of 4.77 Å and a twist of −0.64°. The following commands were used:


relion_image_handler–i 2Hex_221.mrc–new_box 1750–o 2Hex_221_BS1750.mrcrelion_helix_toolbox –impose–i 2Hex_221_BS1750.mrc–o 2Hex_221_BS1750_4.77_t0.64.mrc–cyl_outer_diameter 230–angpix 0.8875–rise 4.77–twist -0.64–z_percentage 0.1


The volume was then processed in Fiji^48^. The central 1,511 slices of the volume (which correspond to the number of slices that generate a single crossover distance) were analysed to visualize the projection. To visualize the full 360° rotation pattern, the images were rotated by 180°, and the 2 projection images were combined.

### Simulation details

Simulations were performed using the GROMACS 2021.7^57^ molecular dynamics package in the isothermal–isobaric NPT ensemble. The temperature was imposed using a velocity rescale thermostat^58^ at 298.15 K with a time constant of 1.0 ps^−1^. The pressure was controlled at 1.0 bar using the Parrinello–Rahman barostat^59^ with a time constant of 2.0 ps^−1^. The equations of motion were integrated using the leap-frog algorithm with a time step of 1.0 fs. Peptide chains and sodium, potassium and chloride ions were modelled using CHARMM36m force-field parameters^60^, and water was described by the TIP3P model^61,62^. The H_2_PO_4_^−^ and HPO_4_^2−^ ions parameters were taken from CHARMM FF on CHARMM-GUI^63,64^. Electrostatic interactions were treated using the smooth particle mesh Ewald method^65^. The solvent molecules and ions were equilibrated by the steepest descent energy minimization with a tolerance of 100.0 kJ mol^−1^, with position restraints in all peptide atoms, followed by 25 ns of molecular dynamics simulation with position restraints only in the backbone atoms of the peptides. After the equilibration of the solvent, the systems were simulated for 100 ns without restraints. All simulations were performed on RAVEN HPC at Max Planck Computing and Data Facility, Garching, Germany.

### Molecular dynamics analysis

All analyses were performed with the GROMACS 2021.7 molecular dynamics package^53^. The RMSF was calculated by residue and averaged over time and layer. The size-independent comparison of the 3D structures was done using the scaled dissimilarity index proposed by Maiorov and Crippen^66^. The trajectories were least-squared fitted to the first snapshot of the simulation, using its backbone atoms as reference. The visualization and simulation snapshots were generated with visual molecular dynamics^67^.

### DILT1 fibril integrity and solvent interaction

A DILT1 fibril in explicit solvent, consisting of 94 layers with each layer rotated by −0.64° (total twist approximately 60°) was simulated to investigate its integrity. The fibril extends across the 22.0 × 22.0 × 44.8 nm^3^ simulation box along the *z* direction, forming an infinitely periodic fibril owing to periodic boundary conditions. The system composition was determined using the systematic equilibrium treatment described previously. The final composition includes: 1,692 peptide chains, 4,739 Cl^−^, 18 H_2_PO_4_^−^, 96 HPO_4_^2−^, 44 K^+^, 1,521 Na^+^, 14,637 DMSO and 509,721 H_2_O.

### Composition of the system for molecular dynamics

The integrity of the DILT1 junction and cross-β-motif was studied by simulating 1-, 3-, 5-, 10- and 15-layer models for each motif. The same number of layers was simulated to verify the conformational persistence of the hexagon fibril. The layer structures were generated by rotating and translating the fundamental junction unit along the *z* direction. The geometric centre of the peptides was placed in a 22.0 × 22.0 × 22.0 nm^3^ simulation box and solvated with a 9:1 (% v/v) mixture of PBS and DMSO.

The system composition was determined from equilibrium calculations at 298.15 K based on the procedure described in ref. ^68^. The dissociation constants of ionizable amino acids (for example, lysine, p*K*_a_ = 10.82) were assumed to be independent of each other. Activity coefficients were computed using the temperature-dependent extended Debye–Hückel equation^68^. The experimental value of the dielectric constant of the water/DMSO mixture, needed to obtain the system composition, was obtained from ref. ^69^, corresponding to 78.08 at 298.15 K.

The equilibrium composition of each system was computed numerically using the Newton–Raphson method, with the change in pH, ΔpH <10^−7^, as the convergence criterion.

### Structure–property relationship simulations

As we have observed that 15 layers are a persistent fibril fragment for DILT1, we have generated samples for the other sequences, DILT2–DILT5, and P1 and P4, with this number of layers. Once the peptide sequence has been mutated in PyMOL, systems were built as before, starting from a junction and applying the corresponding symmetry operations, and simulated following the protocol described above.

### DILT 1 nanofibril characterization

The incubation times and temperatures were varied as indicated below for kinetics and stability (solvent, temperature, sonication) characterizations as follows. (1) Preformed fibrils (1 mg ml^−1^, 10% v/v DMSO, 90% v/v PBS) were obtained by incubating at 22 °C for 24 h with 500 rpm shaking (Extended Data Fig. 7a). (2) Nanofibril formation without pre-dissolved DMSO peptide stock was determined by dissolving the lyophilized peptide directly in DPBS (pH 7.4) yielding a 1 mg ml^−1^ solution (Extended Data Fig. 7b). (3) A dilution stability test was performed on the preformed fibrils as described in (1), which were incubated at 37 °C for 1 week. The fibrils were then diluted in water. Specifically, 10 μl of the preformed fibrils (1 mg ml^−1^) was added to 90 μl Milli-Q H_2_O (0.1 mg ml^−1^) with a final composition of 1% v/v DMSO, 9% v/v PBS and 90% v/v Milli-Q H_2_O. The sample was incubated for 3 days at 37 °C (Extended Data Fig. 7c). This sample was used for the reconstruction of the 1-1-1 morphology (#2; Extended Data Table 1). (4) Early kinetics experiments were conducted by inducing nanofibril formation via the general method with incubation (22 °C, 500 rpm) for either 30 min, 45 min, 1 h, 3 h, 6 h, 9 h, 15 h, 21 h or 24 h (Extended Data Fig. 7k–s). (5) Late kinetics experiments were conducted using the preformed fibrils as described in (1) and transferring them into a 37 °C oven without shaking. The samples were then imaged after 4 days, 1 week, 2 weeks and 53 days (Extended Data Fig. 7t–w). The 53-day sample was used for reconstruction of the 3-3-3 morphology and for the polymorphism analysis (Extended Data Fig. 2). (6) Temperature stability tests were performed by using the preformed fibrils as described in (1) and transferring them for 15 min or 60 min in an oven at 60 °C, 85 °C or 95 °C (Extended Data Fig. 7d–i). (7) Sonication stability tests were performed by exposing preformed fibrils as described in (1) to sonication (15 min, sonication bath) (Extended Data Fig. 7j).

To minimize contamination during long-term incubation, peptide assemblies were prepared from fresh stock solutions using sterile-filtered buffer and clean consumables; full-grid cryo-EM mapping of long-incubation samples did not reveal contaminating micrometre-scale objects.

### Atomic force microscopy

For atomic force microscopy (AFM) screening of DILT1–DILT5 and P3 samples were prepared by introducing the pre-dissolved peptide stock solution (10 mg ml ^−1^, DMSO) to DPBS (pH 7.4), yielding a 1 mg ml^−1^ solution, and analysed after overnight incubation (500 rpm, 25 °C, DPBS, pH 7.4). For imaging the DILT and P3 architectures, a Bruker Dimension FastScan BioTM atomic force microscope was used in the liquid state, which was operated in PeakForce mode. FastScan-D tips from Burker with a nominal spring constant of 0.25 Nm^−1^ were used.

For AFM sample preparation, the pre-incubated peptide solution (100 µl, 1 mg ml^−1^) was added to a circular mica substrate (20 mm) and incubated for 15 min. The excess liquid was removed, washed with 200 µl buffer (DPBS, pH 7.4), excess liquid was removed and 300 µl buffer was added to the mica to measure in liquid. Images were analysed with NanoScope Analysis 1.9.

### In situ humidity-controlled FTIR experiments

Nanofibril formation was induced either by dissolving the DILT1 solid powder directly in the DPBS to yield a 1 mg ml^−1^ solution (DILT1, Fig. 6e and Extended Data Fig. 9b,e–h) or, alternatively, peptide nanofibril formation was induced as described above by introducing the pre-dissolved peptide stock solution (10 mg ml^−1^, DMSO) to DPBS (pH 7.4), yielding a 1 mg ml^−1^ solution (DILT 5, Fig. 6e; DILT 2, Extended Data Fig. 9c; DILT 4, Extended Data Fig. 9d). The same protocol was applied for the control-peptide CKFKFQF. After overnight incubation (500 rpm, 25 °C), DILT1, DILT2, DILT4, DILT5 and CKFKFQF samples, were submitted to in situ humidity-controlled FTIR experiments, respectively. FTIR spectra were recorded in transmission mode using a Bruker VERTEX 70 spectrometer. The spectrometer was purged with nitrogen and all measurements were conducted at room temperature. The pre-incubated peptide samples were loaded into a Teflon flow cell fitted with CaF_2_ windows (1 mm thickness) and a 2 mm path length; the sample (approximately 8 µl) was applied with a thickness ≤0.1 mm. The flow cell was mounted in the spectrometer sample compartment and connected to a dry-nitrogen supply to initiate and control dehydration. Spectra were recorded at regular intervals throughout the drying process, which proceeded until no further spectral change indicated complete desiccation.

### Estimation of the number of water molecules per peptide

The number of water molecules per peptide was estimated using the known infrared absorption cross-sections of the respective functional groups. Molar extinction coefficients of *ϵ*_N–H_ = 100 M^−1^ cm^−1^ for the peptide N–H (ref. ^70^) and *ϵ*_O–H_ = 400 M^−1^ cm^−1^ for the O–H stretch of water molecules^71^ were applied. Considering four N–H groups per peptide and two O–H bonds per water molecule, the integrated intensity ratio satisfies $\frac{{I}_{{\rm{N}}-{\rm{H}}}}{{I}_{{\rm{O}}-{\rm{H}}}}\approx \frac{{N}_{{\rm{N}}-{\rm{H}}}\times {{\epsilon }}_{{\rm{N}}-{\rm{H}}}}{2{N}_{\mathrm{water}}\times {{\epsilon }}_{{\rm{O}}-{\rm{H}}}}$, where *N*_N–H_ is the number of peptide N–H groups and *N*_water_ is the number of water molecules.

For DILT1, after about 110 min in dry nitrogen, the N–H peak at 3,277 cm^−1^ shows an integrated intensity of 0.15, and the broader water O–H band centred at 3,300 cm^−1^ shows an integrated intensity of 7.5, corresponding to approximately 25 water molecules per peptide, assuming negligible contribution from backbone N–H groups (Extended Data Fig. 9b). Considering the channel geometry (0.48 nm peptide-layer height, 6 peptides per layer, 5 nm channel diameter) and a water density of 1.05 g cm^−3^ from simulations, a fully filled channel would contain about 55 water molecules per peptide. The FTIR-derived estimate is inherently approximate, as it relies on extinction coefficients from related systems and does not account for peptide side-chain O–H or N–H contributions. Nevertheless, the number inferred from the infrared spectral intensities is consistent with fully filled tubes at about 100 min drying time inferred from the convergence of the spectral shape at that time.

### In situ dehydration Raman spectroscopy and Cryo-EM experiments

In situ Raman spectra were recorded using a WITec alpha300 Raman microscope equipped with a 532-nm excitation laser. The laser power at the sample was set to 5 mW. The pre-incubated peptide samples (1 mg ml^−1^, directly dissolved in DPBS, 500 rpm overnight shaking, 25 °C) were loaded into a Teflon flow cell fitted with CaF_2_ windows (1 mm thickness) and a 2 mm path length; the sample, approximately 8 µl, was applied with a thickness ≤0.1 mm. The flow cell was fixed on the microscope sample stage and connected to a dry-nitrogen supply, allowing dehydration to be initiated and controlled during Raman acquisition. Spectra were recorded at regular intervals throughout the drying process until no further spectral changes were observed, indicating complete desiccation.

For cryo-EM analysis after controlled drying, a similarly pre-incubated peptide sample was dehydrated using the same Teflon flow-cell and dry-nitrogen protocol as used for the Raman and FTIR experiments. After approximatey 120 min of drying, the flow cell was opened and a TEM grid was dripped into the residual sample liquid on the CaF_2_ window and the TEM grid was then immediately plunge-frozen using a Vitrobot Mark V for subsequent cryo-EM examination.

### Characterization of concentration-dependent self-assembly of DILT1

#### Sample preparation and incubation

DILT1 solutions of various concentrations were prepared. For this, a DILT1 10 mg ml^−1^ DMSO stock solution, prepared in filtered DMSO, was diluted to yield DILT1 DMSO stock solutions at varying concentrations. Subsequently, these pre-dissolved DILT1 DMSO stock solutions were introduced to DPBS (pH 7.4) in a 1:9 ratio, respectively, yielding solutions with varied concentrations (1,000 µg ml^−1^, 500 µg ml^−1^, 100 µg ml^−1^, 50 µg ml^−1^, 35 µg ml^−1^, 17.5 µg ml^−1^, 8.8 µg ml^−1^, 4.4 µg ml^−1^, 2.2 µg ml^−1^, 500 µl, 10% v/v DMSO in DPBS). The respective samples were mixed by brief vortexing and incubated on a shaker (500 rpm, room temperature) overnight. The given values were accurately rounded up to the full number in Extended Data Fig. 8.

#### Vial flip test

After overnight incubation of the DILT1 samples (2–1,000 µg ml^−1^, 10% v/v DMSO in DPBS, 500 µl), the macroscopic gelation behaviour of DILT1 at varied concentrations was examined by vial flip test.

#### Proteostat assay

The critical aggregation concentration of the DILT1 peptide was examined via the commercial Proteostat protein aggregation assay kit by Enzo Life Sciences. Following the manufacturer’s recommendation, adapting a previously published protocol^72^, the Proteostat working solution was prepared by diluting Proteostat stock solution (0.50 µl) and assay buffer (1 µl 10x assay buffer) in 98.5 µl Milli-Q water. To conduct the assay, 36 μl pre-incubated DILT1 peptide solutions at various concentrations (2–1,000 µg ml^−1^, 10% v/v DMSO in DPBS) were mixed with 4 μl Proteostat working solution. The samples were transferred into a Greiner 384 flat black well plate (3 wells per sample (technical replicates *n* = 3), that is, per peptide concentration, 9 µl sample per well), incubated in the dark (15 min) while shaking, and the fluorescence intensity of the Proteostat dye was determined *λ*_excitation_ = 550 nm, *λ*_emission_ = 600 nm; bandwidths 20 nm, multiple reads per well, respectively. At high DILT1 concentration (956 µM), the aggregation affected sample loading to a maximum of 2 wells, each containing 9 µl of the mix (*n* = 2).

#### Transmission electron microscopy

To analyse the aggregate and assembly morphologies at various concentrations, the incubated DILT1 sample solutions (2–1,000 µg ml^−1^, 10% v/v DMSO in DPBS) were submitted to TEM analysis. Grid preparation was carried out as follows. Five microlitres of the sample solution was deposited onto a TEM grid and allowed to stand for 5 min to enable adequate adsorption. Excess solution was then gently removed using filter paper. Subsequently, staining was performed with 5 µl of 4% w/v aqueous uranyl acetate for 2 min. After staining, the grids were rinsed three times with Milli-Q water to remove excess stain. Finally, the water was gently removed from the grid with filter paper, and the grids were left to dry overnight before TEM measurement. TEM was measured at 120 kV.

### Hydrogel and honeycomb fibril preparation

Hydrogels were prepared by either pre-dissolving the DILT1 peptide in DMSO (10 mg ml^−1^) and adding this DMSO peptide stock solution into DPBS to yield a 1 mg ml^−1^ solution (0.1 wt%) or by dissolving solid DILT1 powder directly in the respective solvent to yield 1–4 wt% hydrogels. For example, 0.3 mg peptide was dissolved in 30 µl buffer (for example, DPBS, pH 7) and mixed for approximately 5 s to yield a 1 wt% (10 mg ml^−1^) hydrogel. Very soft hydrogels were obtained at 0.1 wt%, for example, by the nanofibril-formation procedure described in ‘Peptide nanofibril formation’.

### Rheology

Rheological characterization was conducted using a DHR3 rheometer (TA Instruments) equipped with a temperature controller and a solvent reservoir to prevent hydrogel drying. Experiments were performed using an 8-mm parallel-plate geometry with hydrogels of approximately 30 μl volume (gap size of about 0.5 mm). Characterization of the hydrogel mechanical properties was conducted at 25 °C. Gels were prepared directly on the plate by the procedure described in ‘Hydrogel and honeycomb fibril preparation’. Oscillatory time-sweep measurements monitored the gelation at a fixed strain of 0.1% and a fixed frequency of 1 Hz. Extended Data Fig. 8e presents representative sections after complete gelation (approximately last 300 s of gelation curves), which summarizes individual measurements recorded with time stamps at approximately 6-s intervals (time stamps are indicative). Oscillatory strain sweeps (0.01–200% or 0.01–1000%, as indicated) were conducted at a fixed frequency of 1 Hz. Oscillatory frequency sweeps (0.05–100 Hz) were performed at a fixed strain of 0.1%.

### DILT1 gel stability

Photographs were taken with a Canon EOS 250D with a Canon EF 24–105 mm *f*/4L IS lens. Manual mode, exposure of 1/50 with aperture of *f*/8 and focal length of 105 mm. ISO 100. Saved in jpg format with a resolution of 5,284 dpi.

## Online content

Any methods, additional references, Nature Portfolio reporting summaries, source data, extended data, supplementary information, acknowledgements, peer review information; details of author contributions and competing interests; and statements of data and code availability are available at https://doi.org/10.1038/s41586-026-11016-2.

## Supplementary information

Supplementary Information Supplementary Figs. 1–3.

## Figures and Tables

**Extended Data Table 1:**
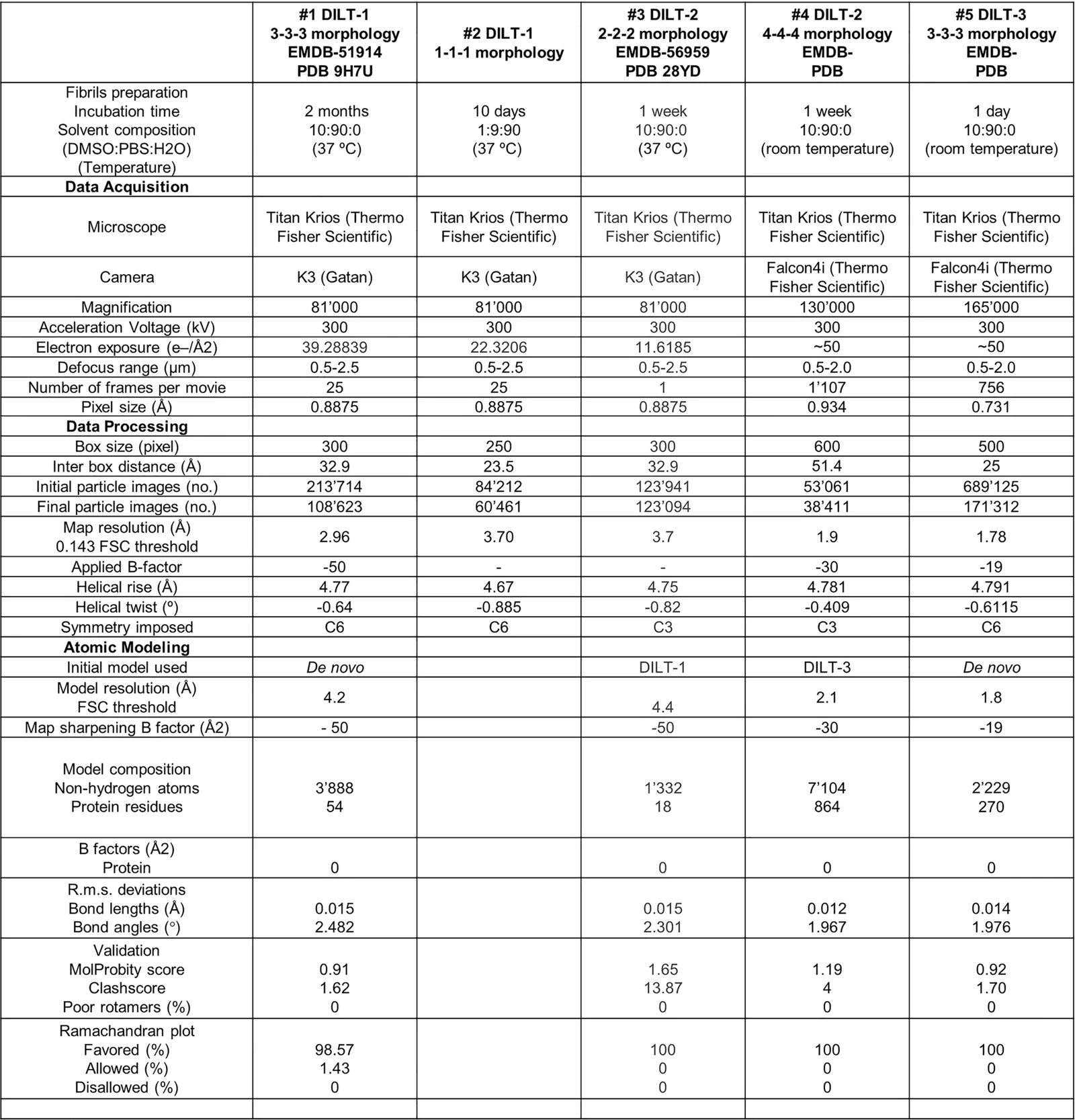
Refinement statistics for the DILT1-DILT3 and their respective 3-3-3 and 1-1-1 (DILT1), 2-2-2 and 4-4-4 (DILT2), as well as 3-3-3 (DILT3) isomorphologies and overview of experimental conditions for DILT1 highlighting the parameters that enable fibril formation

a, Microscope setup for image acquisition and helical parameters used for helical reconstruction. Parameters deriving from the validation tool (MolProbity) for the full atomic model (eighteen chains and three layers) in the DILT1 3-3-3 morphology reconstructed volume.

## Data Availability

The authors declare that the data supporting the findings of this study are available within the paper and its Extended Data files. Data that further support the findings of this study are available from the corresponding authors upon reasonable request. Cryo-EM maps of all unique structures have been deposited in the Electron Microscopy Data Bank (EMDB). Refined atomic models in maps with sufficient resolution for atomic modelling have been deposited in the Protein Data Bank (PDB). The accession numbers (Extended Data Table 1) are EMDB-51914 and PDB 9H7U for DILT1, EMD-56959 and PDB 28YD for DILT2 2-2-2, EMD-56939 and PDB 28XH for DILT2, and EMD-56938 and PDB 28XG for DILT3. Cryo-EM raw data were deposited on EMPIAR with the accession code EMPIAR-13663 (DILT2) and EMPIAR-13687 (DILT3). A patent application covering sequences as well as related applications was filed by the Max-Planck-Gesellschaft (EP26197462.0, inventors are J.G., F.M., I.L., K.L. and T.W.).

## Code availability

GROMACS 2021.7^57^ was used to analyse the simulation trajectories. Custom analysis scripts used in this study are available from the corresponding authors upon reasonable request.
