# Supplementary material for: Sequence-encoded hexagonal lattices in multichannel peptide nanofibrils
Source: Nature. 2026 Sep 23;657(8133):935–43. doi: 10.1038/s41586-026-11016-2 (PMC13600947; doi:10.1038/s41586-026-11016-2)
Supplement: Supplementary file 1 [file 41586_2026_11016_MOESM1_ESM.docx]

**Sequence-Encoded Hexagonal Lattices in Multichannel Peptide Nanofibrils**

*Jasmina Gačanin^1#^, Francesca Mazzotta^1#^, Luis Andre Baptista^1^, Nikolay Stoyanov^2^, Matthias Schmidt^2^, Nico Alleva^1^, Thunchanok Thummaraj^1^, Fanny Bonnicel^1^, Cong Zhou,^1^ Lei Gao, ^1^ Jan Münch^3^, Mischa Bonn,^1^ Marcus Fändrich,^2^ Ingo Lieberwirth,^1^ Robinson Cortes-Huerto^1^, Katharina Landfester^1*^, Tanja Weil^1*^*

*^1^Max Planck Institute for Polymer Research, Ackermannweg 10, 55128 Mainz, Germany*

*^2^Institute of Protein Biochemistry, Ulm University, Helmholtzstr. 8/1, 89081 Ulm, Germany*

*^3^Institute of Molecular Virology, Ulm University Medical Center, Meyerhofstrasse 1, 89081 Ulm, Germany*

*^#^* These authors contributed equally to this work

Supporting Information

Table of Contents

[**Supporting Information Figure 1**	3](#_Toc235173838)

[**Supporting Information Figure 2**	4](#_Toc235173839)

[**Supporting Information Figure 3**	4](#_Toc235173840)

**Supporting Information Figure 1 | DILT1 MALDI mass spectrometry. a,** MALDI-TOF mass spectrum of KVKVSQINM. Measurements were carried out on a Bruker rapifleX^TM^ MALDI-TOF/TOF mass spectrometer using an α-cyano-4-hydroxycinnamic acid matrix. m/z_calc_. = 1045.60, m/z_found_: 1046 [M+H]^+^, 1062 [M+O+H]^+^, 1068 [M+Na]^+^, 1084 [M+K]^+^, 1100, 1106. **b,** MSMS sequencing of peptide KVKVSQINM by MALDI mass spectrometry; precursor amino acid sequence and legend for series (red: b-series, green: y-series)**.** **c,** Calculated and measured fragments of b- and y-series. MALDI matrix: sDHB; solvent: ACN/H_2_O + 0.1% FA Precursor ion: 1046 Da.

**Supporting Information Figure 2 | P2 MALDI mass spectrometry.** MALDI-TOF mass spectrum of KLKLSQINM. Measurements were carried out on a Bruker rapifleX^TM^ MALDI-TOF/TOF mass spectrometer using an α-cyano-4-hydroxycinnamic acid matrix. m/z_calc_. = 1073.63, m/z_found_: 1074 [M+H]^+^, 1090 [M+O+H]^+^, 1096 [M+Na]^+^, 1112 [M+K]^+^, 1128, 1134, 1150, 568.

**Supporting Information Figure 3 | Molecular dynamics stability analysis of fibril constituent. a,** Cylindrical density profile of the principal species in solution (water: blue, chloride ions: green) deriving from the simulation of 94-layer hexagon. The shaded areas correspond to the average position of the fibril atoms. Water density is higher inside compared to outside the fibril. The charged species distribute in such a way that the electrostatic potential remains constant, with DMSO molecules outside the fibril. **b,** Chloride ions are located preferentially inside the hexagon, with an increased density in the surface proximity of the fibrils by interacting with the lysine residues.
